# Detecting electrophysiological alterations in psychiatric disorders through event-related microstates: a systematic review

**DOI:** 10.3389/fpsyt.2025.1591079

**Published:** 2025-09-17

**Authors:** Andrea Perrottelli, Francesco Flavio Marzocchi, Giorgio Di Lorenzo, Chiara D’Amelio, Noemi Sansone, Luigi Giuliani, Pasquale Pezzella, Edoardo Caporusso, Antonio Melillo, Giulia Maria Giordano, Paola Bucci, Armida Mucci, Silvana Galderisi

**Affiliations:** ^1^ Department of Psychiatry, University of Campania “Luigi Vanvitelli”, Naples, Italy; ^2^ Laboratory of Psychophysiology and Cognitive Neuroscience, Department of Systems Medicine, Tor Vergata University of Rome, Rome, Italy; ^3^ IRCCS Fondazione Santa Lucia, Rome, Italy; ^4^ Department of Biotechnological and Applied Clinical Sciences, University of L’Aquila, L’Aquila, Italy

**Keywords:** electroencephalogram (EEG), event-related potentials (ERP), microstates (MS), source localization, mental disorders, neurodevelopmental disorders, psychiatry

## Abstract

**Introduction:**

Event-related potentials (ERPs), recorded through electroencephalography (EEG) during sensory and cognitive tasks, have been consistently employed to investigate electrophysiological correlates of psychiatric disorders. However, traditional peak component analysis of ERPs is limited by the *a priori* selection of time windows and electrodes. Microstate analysis, a data-driven approach based on identifying periods of quasi-stable scalp topographies, has been applied to ERP data, offering a valuable tool for understanding the temporal dynamics of large-scale neural networks. This review aims to provide a comprehensive summary of studies examining event-related microstates in individuals with psychiatric disorders.

**Methods:**

A systematic review of English-language articles indexed in PubMed, Scopus, and Web of Science (WoS) was conducted on May 1, 2024. Studies were included only if they applied microstate analysis to ERP data and analyzed data from at least one group of patients with psychiatric disorders in comparison to healthy controls.

**Results:**

Of the 1,115 records screened, 17 studies were included in the final qualitative synthesis. The majority of these studies (n=8) included patients with schizophrenia, using various tasks focusing mainly on visuospatial processing (n=6) and face processing (n=6). Regarding the microstate methodology, the primary clustering approach employed was the k-means clustering algorithm (n=8), while the cross-validation criterion (n=10) was the most commonly used measure of fit. Sixteen of the 17 studies reported at least one significant difference in microstate features between patients and healthy controls, mainly in the temporal and topographic characteristics of microstates and the sequence of their occurrence.

**Conclusions:**

This review highlights the value of event-related potential microstates analysis in identifying spatiotemporal alterations in brain dynamics associated with psychiatric disorders. However, the limited number of studies and the heterogeneity of experimental paradigms constrain the generalizability of the findings.

**Systematic review registration:**

https://www.crd.york.ac.uk/PROSPERO, identifier CRD42024529185.

## 1 Introduction

Psychiatric disorders are multifactorial conditions characterized by diverse and complex neurobiological alterations (1–5). According to the World Health Organization (6), approximately 1 in every 8 people worldwide experiences a psychiatric disorder, resulting in a significant burden for affected individuals, their careers, and the healthcare systems (6–18). Since their discovery nearly a century ago, event-related potentials (ERPs), measured through electroencephalography (EEG), have been extensively employed to investigate neurobiological alterations associated with psychiatric disorders (19–25). ERPs are positive or negative deflections in brain activity triggered by sensory stimuli or cognitive and motor tasks, recorded within specific time windows after the event of interest (26–28). Most studies analyze the features of the ERPs waveforms, focusing on the maximal voltage amplitude and timing of their peaks. Studies have consistently reported alterations in the amplitude and latency of ERP in individuals with psychiatric disorders. For example, P50, P100, and N100, which are ERP that occur between 50 and 120 milliseconds after stimulus presentation in visual and auditory tasks and reflect early sensory processing, exhibit reduced amplitude in patients with schizophrenia (29–32). Alterations in P300, an EEG index related to attention allocation, working memory, and decision-making, have been observed in individuals with depressive disorders (33), schizophrenia (34), alcohol use disorder (AUD) (35–37), and adults with attention-deficit/hyperactivity disorder (ADHD) (38, 39).

Despite its utility, peak component analysis of ERPs, which generally focuses on the amplitude and latency of their peaks, has several limitations (40). First, the analysis is often restricted to predefined time windows, which significantly limits the amount of information that can be extracted from the recordings. Second, the analysis typically focuses on data from a single electrode or a limited set of electrodes, which reduces its capacity to capture large-scale brain dynamics. Third, the selection of reference electrodes can substantially influence the results, potentially obscuring subtle electrophysiological variations and masking the spatiotemporal complexity of brain electrical activity dynamics (41–43).

An alternative approach to address these limitations is to use EEG microstate (MS) analysis. Microstates are brief periods (generally ranging between 40 and 120 milliseconds) of quasi-stable topographical configurations of scalp potential fields (44–47). This method examines the distribution of electric fields across multiple electrodes to characterize the global electrophysiological state (48). The microstates are considered to reflect the global neuronal activity associated with the activation of distinct brain networks (48). Initially, microstates were identified by analyzing temporal sequences of scalp potential maps recorded during resting-state EEG (44). During rest, a limited number of topographic configurations dominate the temporal series, and studies have identified four to seven canonical MS classes (A, B, C, D, E, F, G) that explain most of the variance in EEG data across healthy subjects and different clinical populations (49–51). For example, MS A is characterized by a right frontal-to-left posterior configuration and it has been related to auditory processing and arousal; conversely, MS B is characterized by a left frontal-to-right posterior configuration and it is related to visual-spatial attention (48). Alterations in resting-state MS features, such as their mean duration, coverage and occurrence (measuring the dominance of the MS), contribution (the relative amount of variance in the EEG signal that is explained by a particular MS class), and topography, are consistently observed in patients with psychiatric disorders (45, 52). For example, resting-state EEG studies showed that patients with depressive disorders exhibit reduced duration and occurrence of MS D (53), which is characterized by a fronto-central configuration, and it has been associated with working memory, while patients with schizophrenia show topographic changes and reduced duration of MS D (50). MS D alterations have also been linked to the severity of positive symptoms in schizophrenia (50), while the relative contribution of microstate A correlates with the severity of negative symptoms (54). Additional findings have also been reported in other conditions. For instance, individuals with autism spectrum disorder (ASD) show reduced duration and coverage of microstate C alongside increased duration and coverage of microstate B (55–57). In ADHD, an increased duration of microstate D has been noted (58, 59).

Studies that have applied MS analysis also to task-based paradigms showed that MS analysis can offer several advantages over traditional peak component analysis of ERPs. In fact, it is a reference-free method that captures rapid (event-related microstates can be even shorter than 40 milliseconds), large-scale brain network dynamics without relying on predefined time windows, making it well-suited to detect subtle neuronal activity changes. Furthermore, MS analysis allows not only the extraction of quantitative parameters, as in peak component analysis of ERPs, to describe the intensity (e.g., mean global field power or area under the curve) and temporal features (e.g., duration, frequency of occurrence, coverage) of microstates, but also the assessment of qualitative features. These include the presence or absence of a specific microstate, its topographical shape, and the order of map appearance, which can provide further details of potential neurophysiological relevance (45, 60). Consequently, over the last two decades, MS analysis has emerged as a valuable tool for analyzing ERPs data, integrating both spatial and temporal features of brain activity (61–64). Furthermore, combining MS analysis with EEG source localization allows researchers to map electrical activity in three dimensions within the cerebral cortex (56, 65–67).

While previous systematic reviews and meta-analyses have focused on resting-state microstate alterations in psychiatric disorders (45, 68–70), the study of alterations of event-related microstates in these pathological conditions remains limited. One recent systematic review investigated MS features in ERPs in patients with psychiatric disorders, but it included only studies using face-processing tasks (71).

One challenge in retrieving and summarizing these studies is that, unlike resting-state microstates, the number, the topography and the temporal characteristics of event-related microstates depend heavily on the specific task performed during EEG recording. This variability complicates the development of a unified nomenclature, as exists for canonical resting-state maps.

Therefore, the present systematic review addresses this gap by examining studies that applied MS analysis to ERPs data in individuals with psychiatric disorders, classifying the results based on the type of task paradigm employed. Specifically, the objectives of this review are to:

1. Describe the demographic and clinical characteristics of the included study samples.
2. Provide an overview of the main EEG preprocessing and MS analysis methodologies.
3. Outline the characteristics of the experimental tasks used to elicit ERPs.
4. Summarize differences in MS features between individuals with psychiatric disorders and healthy controls.
5. Explore the use of source localization data and the associations between MS alterations and clinical features of the included psychiatric disorders.

This systematic review hypothesizes that event-related microstates analysis may capture alterations in the sequences and temporal features of electrophysiological configurations in individuals with psychiatric disorders, offering complementary insights to traditional ERP peak component analysis. We also predict that studies employing similar paradigms may yield comparable topographic microstate maps, enabling cross-study comparisons.

## 2 Materials and methods

### 2.1 Design of the review and search strategy

The review protocol was registered on the International Prospective Register of Systematic Reviews (PROSPERO) under registration number CRD42024529185. The review was conducted in accordance with the updated 2020 PRISMA Statement guidelines (72).

A systematic search for relevant articles was performed in three electronic databases—PubMed, Scopus, and Web of Science (WoS)—on 1st May 2024, without any time restrictions. The objective was to identify studies that employed microstate analysis in event-related potential (ERP) paradigms involving subjects with psychiatric disorders.

The following search string was applied to all databases:

((EEG) OR (electroencephalogra*)) AND (microstate) AND ((schizo*) OR (Psycho*) OR (Bipolar*) OR (Depress*) OR (Anxiet*) OR (Obsessive Compulsive Disorder) OR (Trauma) OR (Dissociative Disorder) OR (Somatic Disorder) OR (Substance-Related Disorder) OR (Addictive Disorder) OR (Eating Disorder) OR (Personality Disorder) OR (Conduct Disorder) OR (OCD) OR (ADHD) OR (mental) OR (autis*) OR (neurodevelopmental disorder) OR (psychiatric disorder)).

### 2.2 Selection process and eligibility criteria

Cohort and case-control studies published in English that included human subjects with at least one group of participants diagnosed with psychiatric disorders (including: Schizophrenia Spectrum and other psychotic disorders; Bipolar Disorder; Depressive Disorders; Anxiety Disorders; Obsessive-Compulsive and Related Disorders; Trauma- and Stressor-Related Disorders; Dissociative Disorders; Somatic Symptom and Related Disorders; Substance-Related and Addictive Disorders; Eating Disorders; Personality Disorders; Disruptive, Impulse-Control, and Conduct Disorders; Autism Spectrum Disorder or Attention-Deficit/Hyperactivity Disorder) and that recorded EEG data during sensory, cognitive, or emotional processing tasks and employed EEG event-related MS analysis comparing data from patients with psychiatric disorders to healthy controls were included in the review.

Publications such as book chapters, comments, editorials, case reports/series, theses, proceedings, letters, short surveys, notes, or studies irrelevant to the aim of the review (e.g., those analyzing either resting-state EEG data or EEG data in populations without psychiatric disorders), or studies for which full text was unavailable or studies focusing exclusively on children aged 9 years or younger were excluded from the review.

Two researchers (F.F.M. and C.D.A.) independently screened all articles based on titles and abstracts to assess eligibility, followed by full-text evaluations. Discrepancies in article selection were resolved through group discussion and consensus. However, given the anticipated high heterogeneity in study paradigms and methodologies, a meta-analysis was not planned.

### 2.3 Data extraction

The following data were extracted from each eligible article: publication details (authors and year of publication), study population (diagnosis, sample size, gender distribution, and age of participants), methodology (inclusion and exclusion criteria; description of the experimental task protocol; EEG recording system used; ERPs analyzed within the MS framework; time window considered for MS analysis; software used for MS analysis; EEG preprocessing details, such as sampling rate, band-pass settings, clustering algorithm; number of MS clusters identified and global explained variance (73); MS parameters considered), results (MS analysis comparing patients and healthy controls; if available, additional analyses, such as peak component analysis or source localization).

For studies reporting peak component analysis data, Cohen’s *d* was calculated to estimate effect sizes for peak- and microstate-related results.

### 2.4 Risk of bias assessment

The risk of bias was evaluated for eligible articles using the Newcastle-Ottawa Scale (NOS) (74), which assesses study quality across three domains: selection (i.e., how well the study selects participants), comparability (i.e., how well the study controls for confounding variables) and outcome (i.e., how effectively the outcomes are measured and reported).

Studies were categorized based on their NOS score (0 to 9) as follows: poor quality/high risk of bias (0–3), fair quality/moderate risk of bias (4–6) and good quality/low risk of bias (7–9).

## 3 Results

**Figure 1:**
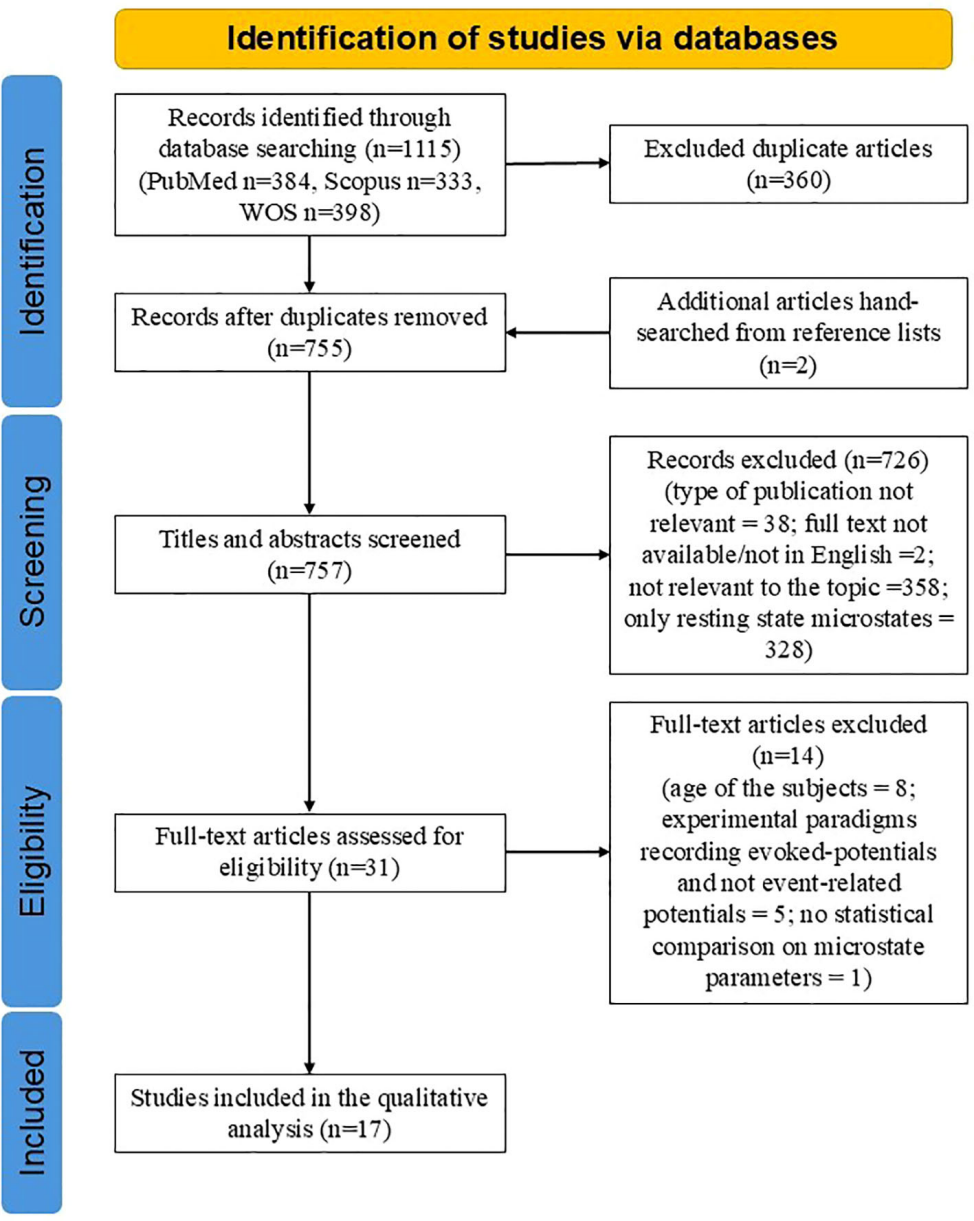
PRISMA flowchart of included studies.

The combined search from three databases yielded a total of 1,115 records. After removing duplicates, 755 articles remained. Two additional articles were identified through manual screening of reference lists, bringing the total to 757 articles. After the abstract screening, 726 articles were excluded for various reasons: they were irrelevant to the topic (e.g., did not use EEG microstates, focused only on resting-state EEG microstates, or lacked subjects with psychiatric disorders), or the full text was unavailable in English (**Figure 1**). Thirty-one full-text articles were assessed, leading to further exclusions: eight articles due to subject age (<10 years), five due to inadequate experimental paradigms, one for not reporting statistical analysis comparing MS parameters between patient and control groups and one for lacking a control group. Ultimately, 17 articles were included in the qualitative analysis (**Figure 1**).

All included studies, except one with a moderate risk of bias (NOS score: 6) (75), had a low risk of bias (NOS scores: 7–9) (**Table 1**).

**Table 1:** Description of the studies included: demographic and clinical characteristics of the samples.

First Author, year	Diagnosis	Sample size and demographic characteristics of the subjects	Inclusion/exclusion criteria of the study	Risk of bias assessment (NOS) (0-9)
Antonova et al., 2021 (76)	Subjects with schizophrenia divided into two groups: patients with auditory verbal hallucination (AVH) and patients without hallucination (NH)	**Total Subjects (n)**: 46 Mean Age (years): 37.8 *AVH group (n): 16* *Mean Age (years): 39.8* *Gender (n. of males): 9* *NH group (n): 10* *Mean Age (years): 37.2* *Gender (n. of males): 8* *HC group (n): 20* *Mean Age (years): 36.5* *Gender (n. of males): 7*	- All groups: right-handed; normal or corrected-to-normal vision - HC group: no history of psychiatric or neurological disorders or head trauma - NH group: patients with no history of auditory hallucinations - AVH group: patients with frequent auditory hallucinations	9
Begré et al., 2008 (77)	Subjects with first episode schizophrenia (FESZ)	**Total Subjects (n)**: 22 Mean Age (years): 24.3 *FESZ group (n): 11* *Mean Age (years): 25.2* *Gender (n. of males): 7* *HC group (n): 11* *Mean Age (years): 23.4* *Gender (n. of males): 7*	- All groups: right-handed - FESZ group: diagnosis of schizophrenia according to ICD-10 at their first episode - HC group: no diagnosis of psychiatric disease, neurological or major medical disorders and substance abuse	9
Berchio et al., 2017 (83)	Female subjects with borderline personality disorder (BPD)	**Total Subjects (n)**: 36 Mean Age (years): 24.2 *BPD group (n): 18* *Mean Age (years): 25.0* *Gender (n. of males): 0* *HC group (n): 18* *Mean Age (years): 23.4* *Gender (n. of males): 0*	- BPD group: BPD diagnosis assessed by SCID-II; no diagnosis of axis I disorders - HC group: no history of psychiatric illness; report no use of medications or substances	7
Berchio et al., 2019 (85)	Subjects with Bipolar Disorder (BD) and their offspring	**Total subjects (n):** 72 Mean Age BD (years): 34.99 *BD group (n): 18* *Mean Age (years): 34.94* *Gender (n. of males): 11* *HC age-matched (n): 18* *Median Age (years): 35.03* *Gender (n. of males): 11* Mean Age Offspring (years): 19.61 *Offspring group (n): 18* *Mean Age (years): 19.72* *Gender (n. of males): 6* *HC age-matched (n): 18* *Median Age (years): 19.50* *Gender (n. of males): 8*	-All groups: speak French, no brain injury or neurological disease -BD: having offspring aged 15–25 years, previous diagnosis of BD I or II, euthymic -HC: no psychiatric illness	8
Chang et al., 2022 (78)	Subjects with first episode schizophrenia (FESZ) and ultra-high-risk individuals (UHR)	**Total Subjects (n)**: 105 Mean Age (years): 24.54 *FESZ group (n): 35* *Mean Age (years): 25.09* *Gender (n. of males): 15* *UHR group (n): 30* *Mean Age (years): 22.67* *Gender (n. of males): 15* *HC group (n): 40* *Mean Age (years): 25.45* *Gender (n. of males): 27*	- All groups: no diagnosis of delirium, dementia or other cognitive disorders, intellectual impairment (IQ ≤ 70); no suicidal ideation or self-harm; no severe physical disease; no electric twitch or magnetic stimulation received within 6 months; no impaired hearing - FESZ group: no other diagnosis of psychiatric disorders - HC and UHR groups: No diagnosis of schizophrenia spectrum disorder, bipolar disorder, brain organic disorder, physical illness or psychoactive disorder	8
Galderisi et al., 2001 (63)	Subjects with a diagnosis of panic disorder (PD)	**Total Subjects (n):** 28 Mean Age (years): 26.3 *PD group (n): 14* *Mean Age (years): 27.4* *Gender (n. of males): 7* *HC group (n): 14* *Mean Age (years): 25.2* *Gender (n. of males): 7*	- All groups: age < 45 years; least 5 years of education; negative neurological examination; no history of mental retardation, organic mental disorders, alcoholism, drug abuse, head injury, and neurological disorders - PD group: a DSM-IV diagnosis of panic disorder; no withdrawal of psychopharmacological treatment at least 15 days before starting the experimental procedure - HC group: no personal or family history of psychiatric disorders	8
Haartsen et al., 2020 (84)	Patients with autism spectrum disorder (ASD)	**Total Subjects (n)**: 305 Mean Age adult group (years): 23.01 Mean Age adolescent group (years): 15.09 *ASD adult group (n): 87* *Mean Age (years): 22.6* *Gender (n. of males): 64* *HC adult group (n): 73* *Mean Age (years): 23.5* *Gender (n. of males): 53* *ASD adolescent group (n): 79* *Mean Age (years): 15.0* *Gender (n. of males): 62* *HC adolescent group (n): 66* *Mean Age (years): 15.2* *Gender (n. of males): 41* *Data on children were not considered*	*-* All groups: IQ > 75; Age: adolescents group = 12-17; adults = 18-31; no significant uncorrected visual or hearing difficulties, no history of alcohol and/or substance use or independence in the past year *-* ASD groups: diagnosis of autism	7
Kleinlogel et al., 2007 (75)	Hospitalized subjects diagnosed with first episode of schizophrenia (FESZ)	**Total subjects (n):** 36 Mean Age (years): 24.8 *FESZ group (n): 18* *Mean Age (years): 25* *Gender (n. of males): 15* *HC group (n): 18* *Mean Age (years): 24.6* *Gender (n. of males): 15*	- FESZ group: ICD-10 diagnosis of schizophrenia - HC group: no history of major medical or neurological disorder, no substance abuse or other psychiatric disease diagnosis or previous psychotropic treatment	6
Kochi et al., 1996 (79)	Subjects diagnosed with schizophrenia (SCZ)	**Total subjects (n):** 26 Mean Age (years): 26 *SCZ group (n): 13* *Mean age (years): 26.7* *Gender (n of males): 13* *HC group (n): 13* *Mean age (years): 25.3* *Gender (n. of males): 13*	- Both groups: no history of head trauma; no history or current drug addiction; no neurological brain disease - SCZ group: optimal antipsychotic treatment - HC group: right-handed; intact color vision	9
Mauriello et al., 2022 (88)	Subjects with ADHD	**Total subjects (n):** 46 Mean Age (years): 23.75 *ADHD group (n): 23* *Mean Age (years): 24.2* *Gender (n. of males): 13* *HC group (n): 23* *Mean Age (years): 23.3* *Gender (n. of males): 13*	- Both groups: no history of head injury or mental retardation; no current drug or alcohol abuse - HC group: no history of psychiatric or neurological disease	8
Mucci et al., 2007 (81)	Subjects with diagnosis of deficit schizophrenia (DS) and non-deficit schizophrenia (NDS).	**Total Subjects (n)**: 60 Mean Age (years): 36.07 *DS group (n): 20* *Mean Age (years): 37.2* *Gender (n. of males): 17* *NDS group (n): 20* *Mean Age (years): 35.3* *Gender (n. of males): 17* *HC group (n): 20* *Mean Age (years): 35.7* *Gender (n. of males): 17*	-Both patients’ groups: diagnosis of schizophrenia assessed by SCID-I; aged 16–55 y; no severe mental retardation, alcoholism, or drug abuse or dependence in the last 12 months; no previous electroconvulsive therapy. -DS group: diagnosis of DS, as assessed by the Schedule for the Deficit Syndrome (SDS) scale -Healthy control group: no personal or family history of major psychiatric disorders; no history of severe head trauma or substance-related disorders.	8
Perizzolo Pointet et al., 2021 (86)	Female subjects with lifetime interpersonal Violence exposure (IPV) (i.e. exposure to domestic violence, physical and/or sexual abuse, among other life events) related post-traumatic stress disorder (IPV-PTSD).	**Total Subjects (n)**: 30 Mean Age (years): 38.38 *IPV-PTSD group (n): 16* *Mean Age (years): 39.00* *Gender (n. of males): 0* *HC group (n): 14* *Mean Age (years): 37.67* *Gender (n. of males): 0*	- IPV-PTSD group: History of experience of IPV and other traumatic events during childhood and adulthood was assessed using the BPSAQ and the TLEQ respectively. PTSD symptoms were assessed using the CAPS (lifetime symptoms) and the PCL-S (current symptoms prior to one month). PTSD according to DSM IV-TR criteria. - HC group: no PTSD DSM IV-TR, no PTSD symptoms according to CAPS and PCL-S	7
Perrottelli et al., 2023 (64)	Subjects with schizophrenia (SCZ)	**Total Subjects (n)**: 53 Mean Age (years): 33.08 *SCZ group (n): 30* *Mean Age (years): 34.23* *Gender (n. of males): 18* *HC group (n): 23* *Mean Age (years): 31.57* *Gender (n. of males): 10*	- SCZ group: Diagnosis of schizophrenia using MINI-Plus; age: 18-65; no hospitalization or change in psychotropic medication for 3 months before recording; treated only with second-generation antipsychotics; negative neurological examination and no history of moderate intellectual disability, neurological illness, head injury with loss of consciousness, alcoholism or drug abuse or dependence in the last 6 months (except for smoking) - HC group: no mental disorder AXIS I according to MINI-Plus; no familiar history of psychosis or affective disorders	8
Rohde et al., 2020 (42)	Subjects with alcohol use disorder (AUD) during detoxification treatment who had been abstinent for a minimum of 8 days at the start of the study	**Total Subjects (n)**: 30 Mean Age (years): 44.8 *AUD group (n): 15* *Mean Age (years): 46.2* *Gender (n. of males): 12* *HC group (n): 15* *Mean Age (years): 43.4* *Gender (n. of males): 9*	- All groups: no other diagnosis of axis I disorders - AUD group: patients with a diagnosis of alcohol use disorder according to ICD-10	7
Soni et al., 2019 (80)	Subjects diagnosed with schizophrenia (SCZ)	**Total subjects (n):** 56 Mean Age (years): 27.41 *Schizophrenia (SCZ) group (n): 28* *Mean Age (years): 27.21* *Gender (n. of males): 20* *First-degree relatives (FDR) group (n): 28* *Mean Age (years): 31.17* *Gender (n. of males): 18* *HC group (n): 28* *Mean Age (years): 27.76* *Gender (n. of males): 17*	- All groups: no history of neurological injury; no serious medical illness or substance use disorders; right-handed - SCZ group: less than 5 years illness duration; at least 8 years of formal education; no hospitalization from at least 2 months; stable medication regimen with 2^nd^ generation antipsychotics for at least 8-months	9
Tschuemperlin et al., 2020 (82)	Subjects with Alcohol Use Disorder (AUD)	**Total subjects (n):** 83 Mean Age (years): 44.98 *AUD group (n): 62* *Mean Age (years): 43.28* *Gender (n. of males): 40* *HC group (n): 21* *Median Age (years): 50* *Gender (n. of males): N.A.*	- Both groups: no high scores on psychopathology (as assessed through the Brief Symptom Check List); no current treatment/diagnosis for psychiatric comorbidity; no past treatment for substance abuse disorder; no problematic substance use; no AUD in first-degree relatives; no hearing impairment - AUD group: detoxification and abstinence from alcohol for > 23 days; no other severe substance abuse; no neurocognitive disorder diagnosis; no current treatment with benzodiazepines or methylphenidate	9
Zhao et al., 2023 (87)	Adolescents with Major Depressive Disorder (MDD) with nonsuicidal self-injury (NSSI) behavior or without NSSI MDD subjects with NSSI were evaluated before and after rTMS.	**Total Subjects (n)**: 138 Mean Age (years): 14.86 *MDD-NSSI group (n): 66* *Mean Age (years): 14.33* *Gender (n. of males): 12* *MDD group (n): 52* *Mean Age (years): 15.31* *Gender (n. of males): 20* *HC group (n): 20* *Mean Age (years): 15.45* *Gender (n. of males): 8*	- All groups: age 12–17 years; normal or corrected vision, normal hearing; no neurological disorders or other psychiatric disorders; no chronic substance use; No head injuries with loss of consciousness - MDD-NSSI group: patients with a diagnosis of MDD (according to ICD-10) with nonsuicidal self-injury behavior (according to Ottawa Self-Injury Scale) - MDD group: Patients with a diagnosis of MDD (according to ICD-10) without nonsuicidal self-injury behavior - HC group: No MDD or nonsuicidal self-injury behavior	7

ADHD, attention deficit-hyperactivity disorder; ASD, autism spectrum disorder; AUD, alcohol use disorder; AVH, auditory verbal hallucination; BD, bipolar disorder; BPD, borderline personality disorder; BPSAQ, brief physical and sexual abuse questionnaire; CAPS, clinician-administered PTSD scale; CPT, continuous performance test; CRT, choice reaction task; DS, deficit schizophrenia; DSM, Diagnostic and Statistical Manual of Mental Disorders; FESZ, first episode schizophrenia; HC, healthy control; IAT, alcohol valence task; ICD, International Classification of Diseases; IPV, Interpersonal Violence exposure; IQ, intelligence quotient; MDD, major depressive disorder; MID, monetary incentive delay task; MINI, Mini-International Neuropsychiatric Interview; NDS, non deficit schizophrenia; NH, no hallucination; NOS, Newcastle–Ottawa scale; NSSI, non-suicidal self-injury; PCL-S, PTSD Checklist Scale; PD, panic disorder; PTSD, post-traumatic stress disorder; rTMS, repetitive transcranial stimulation; SCID-I/II, Structured Clinical Interview for DSM-IV Axis I/II; SCZ, schizophrenia; SDS, Schedule for the Deficit Syndrome; TLEQ, Traumatic Life Events Questionnaire; UHR, Ultra-High-Risk for psychosis; VSWM, Visuospatial working memory task.

### 3.1 Clinical and demographic characteristics of the samples and experimental tasks

The studies focused on various psychiatric and neurodevelopmental conditions (**Table 1**). Nearly half (n=8) involved patients with schizophrenia (64, 75–81), including three studies of first-episode schizophrenia (75, 77, 78), one of which also included individuals at high risk for psychosis (78) and one study which included patients with deficit schizophrenia (81). Two studies focused on alcohol use disorder (AUD) (42, 82). Other conditions investigated included borderline personality disorder (BPD) (83), autism spectrum disorder (ASD) (84), bipolar disorder (BD) (85), panic disorder (PD) (63), post-traumatic stress disorder (PTSD) (86), major depressive disorder (MDD) (87), and attention-deficit/hyperactivity disorder (ADHD) (88).

The average sample size per study was 68.9 (**Table 1**), ranging from 22 to 305 participants. Two studies involved adolescents aged 10–17 years (84, 87), ten included young adults aged 18–30 years (63, 75, 77–80, 83–85, 88), five included participants aged 31–40 years (64, 76, 81, 85, 86), and two involved participants older than 41 years (42, 82).

The experimental paradigms employed during EEG recordings were highly heterogeneous (see **Table 1** for a detailed description of the tasks). Overall, studies focusing on schizophrenia mainly employed paradigms addressing related cognitive domains related to executive functioning, such as attention and vigilance (75–77, 81), and working memory (80), due to the remarkable impairments that can be traced in these domains in subjects experiencing this disorder, both using clinical evaluations and EEG indices associated with these domains (89–92). The majority of studies involving face processing tasks included patients affected either by mood disorders (MDD and BD) (85, 87) or neurodevelopmental disorders, such as autism and ADHD (84, 88), due to the alterations in EEG correlates recorded in these pathologies during different stages of processing of faces and emotions. Finally, tasks using alcohol-related cues (42, 82) were employed for patients affected by AUD to obtain EEG indices of the neural mechanisms underlying cue reactivity in alcohol craving.

### 3.2 Characteristics of EEG acquisition and microstate analysis

A detailed summary of the EEG preprocessing procedures, MS analysis procedures and MS features considered can be found in **Table 2** and **Table 3**.

**Table 2:** ERP, analysis, software and preprocessing procedures of the studies.

First Author, year	EEG recording system	Time window of the MS analysis	Software for MS analysis	MS preprocessing characteristics
Antonova et al., 2021 (76)	74-electrodes BrainScope EEG system.	0–1000 ms from the onset of the task	RAGU	Sampling rate for analysis (Hz): 250 Band-pass settings (Hz): 0.3 - 70 Notch filter (Hz): 50 ICA correction: Yes Clustering algorithm: AAHC algorithm Evaluation of the optimal number of MS: cross-validation criterion, GEV Analysis for source localization: N/A MS consistency/temporal smoothing during microstate analysis: topographic consistency test
Begré et al., 2008 (77)	21-electrodes BrainScope EEG system.	0–600 ms from stimulus onset	Brain Vision Analyzer	Sampling rate for analysis (Hz): 250 Band-pass settings (Hz): 0.5 - 70 Notch filter (Hz): 50 ICA correction: Yes Clustering algorithm: topographic Microstate Clustering Evaluation of the optimal number of MS: 150 topographical 4 ms-maps for the 600 ms time window of analysis were set *a priori* for all subjects; number of MS picked based on the most frequently observed optimum of MS clusters across subjects cross-validation criterion Source localization method: N/A MS consistency/temporal smoothing during microstate analysis: N/A
Berchio et al., 2017 (83)	256-electrodes Electrical Geodesic Inc. system.	0–500 ms from stimulus onset	Cartool	Sampling rate for analysis (Hz): 1000 Band-pass settings (Hz): 0.3 - 40 ICA correction: No Clustering algorithm: k-means clustering Evaluation of the optimal number of MS: Cross-validation criterion Source localization method: LAURA MS consistency/temporal smoothing during microstate analysis: N/A
Berchio et al., 2019 (85)	256-electrodes Electrical Geodesic Inc. system.	0–400 ms from stimulus onset	Cartool	Sampling rate for analysis (Hz): 1000 Band-pass settings (Hz): 0.3 - 40 ICA correction: No Clustering algorithm: k-means Evaluation of the optimal number of MS: Meta-criterion Source localization method: LAURA MS consistency/temporal smoothing during microstate analysis: N/A
Chang et al., 2022 (78)	128-electrodes Electrical Geodesics Inc. system.	-200–1000 from the onset of the first auditory click (S1)	Microstate EEGlab toolbox	Sampling rate for analysis (Hz): 1000 Band-pass settings (Hz): 1 - 40 ICA correction: Yes Notch filter (Hz): 50 Clustering algorithm: modified k-means cluster analysis Evaluation of the optimal number of MS: GEV and cross-validation criterion Source localization method: eLORETA MS consistency/temporal smoothing during microstate analysis: microstate with a duration of less than 10 ms were rejected
Galderisi et al., 2001 (63)	13-electrodes EASYS2 system.	0–800 ms from stimulus onset	N/A	Sampling rate for analysis (Hz): 100 Band-pass settings (Hz): 0.5 - 35 Notch filter (Hz): 50 ICA correction: No Clustering algorithm: N/A Evaluation of the optimal number of MS: Stability and discrimination criteria (as illustrated by Koenig and Lehmann 1996) Source localization method: N/A MS consistency/temporal smoothing during microstate analysis: N/A
Haartsen et al., 2020 (84)	62-electrodes system (Brand: N/A)	0–800 ms from stimulus onset	RAGU	Sampling rate for analysis (Hz): 1000 Band-pass settings (Hz): 1 - 40 Notch filter (Hz): 50 ICA correction: No Clustering algorithm: atomize and agglomerate hierarchical clustering (AAHC) algorithm Evaluation of the optimal number of MS: cross-validation method, identified as the number of microstates with the highest level of explained variance before which the fit plateaus Source localization method: N/A MS consistency/temporal smoothing during microstate analysis: topographic consistency test and smoothing applied with a window size of 40 and non-smoothness penalty of.3 to suppress very short microstates
Kleinlogel et al., 2007 (75)	21-electrodes BrainScope EEG system	0–600 ms from stimulus onset	Brain Vision Analyser	Sampling rate for analysis (Hz): 250 Band-pass settings (Hz): 12 (high cut-off filter only) Notch filter (Hz): 50 ICA correction: Yes Clustering algorithm: 150 topographical 4 ms-maps for the 600 ms were set *a priori* for all subjects; number of MS picked based on the most frequently observed MS-solution across subjects Evaluation of the optimal number of MS: N/A Source localization method: LORETA MS consistency/temporal smoothing during microstate analysis: N/A
Kochi et al., 1996 (79)	16-electrodes system (Brand: N/A)	0–1000 ms from stimulus onset	N/A	Sampling rate for analysis (Hz): N/A Band-pass settings (Hz): 1 - 30 Notch filter (Hz): 30 ICA correction: No Clustering algorithm: N/A Evaluation of the optimal number of MS: Pearson’s correlations were calculated between the spatial configuration of maps to obtain the Global Map Dissimilarity value Source localization method: N/A MS consistency/temporal smoothing during microstate analysis: N/A
Mauriello et al., 2022 (88)	256-electrodes EGI Philips Electrical Geodesics, Inc. system	0–600 ms from stimulus onset	Cartool	Sampling rate for analysis (Hz): 1000 Band-pass settings (Hz): 0.4 - 40 Notch filter (Hz): 50 ICA correction: No Clustering algorithm: k-means clustering algorithm Evaluation of the optimal number of MS: meta-criterion Source localization method: LORETA MS consistency/temporal smoothing during microstate analysis: MS lasting less than 20 ms were excluded
Mucci et al., 2007 (81)	32-electrodes BrainScope EEG system	0–800 ms from stimulus onset	N/A	Sampling rate for analysis (Hz): 256 Band-pass settings (Hz): 0.5 - 15 Notch filter (Hz): N/A ICA correction: N/A Clustering algorithm: N/A Evaluation of the optimal number of MS: N/A Analysis for source localization: LORETA MS consistency/temporal smoothing during microstate analysis: N/A
Perizzolo Pointet et al., 2021 (86)	256-electrodes Electrical Geodesic Inc. system.	-100–600 ms from stimulus onset	Cartool	Sampling rate for analysis (Hz): 1000 Band-pass settings (Hz): 0.1 - 40 Notch filter (Hz): Yes, value not specified ICA correction: No Clustering algorithm: k-means cluster analysis Evaluation of the optimal number of MS: Krzanowski–Lai criterion Analysis for source localization: LAURA inverse solution model MS consistency/temporal smoothing during microstate analysis: N/A
Perrottelli et al., 2023 (64)	32-electrodes BrainScope EEG system	-500–1000 ms from the cue stimulus onset	RAGU	Sampling rate for analysis (Hz): 256 Band-pass settings (Hz): 0.1 - 70 Notch filter (Hz): 50 ICA correction: Yes Clustering algorithm: k-means clustering algorithm Evaluation of the optimal number of MS: cross-validation criterion (the number of microstates with the highest level of explained variance before which the fit plateaus) Analysis for source localization: sLORETA MS consistency/temporal smoothing during microstate analysis: topographic consistency test and brief microstates were excluded (specific parameter is not stated)
Rohde et al., 2020 (42)	70-electrodes Neurofax 1100G system	0–1000 ms from stimulus offset	RAGU	Sampling rate for analysis (Hz): 500 Band-pass settings (Hz): 0.5 - 18 Notch filter (Hz): 50 ICA correction: Yes Clustering algorithm: modified k-means clustering algorithm Evaluation of the optimal number of MS: GEV and cross-validation criterion Analysis for source localization: N/A MS consistency/temporal smoothing during microstate analysis: topographic consistency test and microstates of at least 60ms duration
Soni et al., 2019 (80)	128-electrodes HydroCel Geodesic Sensor Net system	-50–0 ms from stimulus (pre-trial MS) and response (pre-response MS) onset	Cartool	Sampling rate for analysis (Hz): 1000 Band-pass settings (Hz): 1 - 100 Notch filter (Hz): 50 ICA correction: Yes Clustering algorithm: k-means clustering algorithm Evaluation of the optimal number of MS: GEV and cross-validation criterion Source localization method: LORETA MS consistency/temporal smoothing during microstate analysis: N/A
Tschuemperlin et al., 2020 (82)	64-electrodes BrainVision Recorder	30–1000 ms from stimulus onset	RAGU	Sampling rate for analysis (Hz): 500 Band-pass settings (Hz): 0.5 - 20 Notch filter (Hz): 50 ICA correction: Yes Clustering algorithm: k-means clustering algorithm Evaluation of the optimal number of MS: cross-validation procedure Source localization method: LORETA MS consistency/temporal smoothing during microstate analysis: smoothing procedure of Pascual-Marqui et al. (1995) was implemented to reduce the amount of very small microstates (window of 20 time points or under, smoothness penalty of 3.9) and one remaining microstate under 20ms was excluded from analyses
Zhao et al., 2023 (87)	64-electrodes Churry 8 system	-200–1000 ms from stimulus onset	Microstate EEGlab toolbox	Sampling rate for analysis (Hz): 500 Band-pass settings (Hz): 0.1 – 30 Notch filter (Hz): No ICA correction: Yes Clustering algorithm: k-means clustering algorithm Evaluation of the optimal number of MS: GEV and cross-validation criterion Analysis for source localization: N/A MS consistency/temporal smoothing during microstate analysis: N/A

AAHC, atomize and agglomerate hierarchical clustering; EEG, electroencephalography; GEV, Global Explained Variance; ICA, independent component analysis; LAURA, Local Autoregressive Average; s/eLORETA, standardized/exact Low-Resolution Electromagnetic Tomography; MS, microstate;

**Table 3:** Main results of the MS analysis.

First Author, year	Experimental task	Number of microstates retrieved (with associated ERP and onset and offset and, if specified) and global explained variance	MS parameters considered	Results
Antonova et al., 2021 (76)	*4-Choice Reaction Task (4-CRT) with lateralized stimuli:* The experiment involved participants viewing four white square outlines on a black background. Each trial featured one white square filling for 100 ms at random positions. Participants fixed their gaze between the middle squares and responded by pressing keys on a response board corresponding to the target position, (Left: LL; left middle ML; right middle MR; and right RR). The task comprised four blocks of 72 trials each.	7 Microstates: - MS 1: right lateralized N1 (112 – 248 ms) - MS 2: left lateralized N1(112 – 248 ms) - MS 3: central P3a - MS 4: parietal P3b - MS 5: Post P3 activity - MS 6: frontocentral negativity and parietal positivity after response (for HC group) or during response (AVH NH) - MS 7: Post P3 activity GEV learning sets: 82% GEV test sets: 65%	- Duration - AUC	*Duration* - MS 3: HC<AVH<NH (p=0.049) - MS 3: HC<patients (p=0.003) - MS 3: HC<AVH (p=0.006) - MS 3: HC<NH (p=0.029) - MS 6: AVH<NH<HC (p=0.008) - MS 6: HC>patients (p=0.0008) - MS 6: HC>AVH (p=0.0002) - MS 6: HC>NH (p=0.068) *AUC* - MS 1: HC>patients (p=0.002) - MS 1: HC>AVH (p=0.008) - MS 3: HC<AVH<NH (p=0.079) - MS 3: HC<patients (p=0.012) - MS 3: HC<AVH (p=0.01) - MS 3: HC<NH (p=0.041) - MS 4: NH<HC<AVH (p=0.053) - MS 4: AVH>NH (p=0.032) - MS 4: HC>NH (p=0.096) - MS 5: AVH=NH=HC (p=0.67)
Begré et al., 2008 (77)	*Continuous performance test (CPT):* Twelve different letters were presented in random order on the screen. Each letter was presented for 200 ms in the center of the screen with an inter-trial interval of 1450 ms. The letter O (cue) was presented as a signal to prepare a motor response (primer condition). Subjects were instructed to press a button only when the cue letter was followed by the target letter X (Go stimulus). The other letters A-H, J, and L thus required response inhibition if they immediately followed an O (NoGo-stimulus).	1 Microstate: - MS P300 (for both Go/NoGo Stimuli) (time window N/A) GEV: N/A	- Duration	*Duration* - MS P300 in Go Stimuli: no significant difference between SCZ and HC (p > 0.05) - MS P300 in NoGo Stimuli: no significant difference between SCZ and HC (p > 0.05)
Berchio et al., 2017 (83)	*2-back gaze working memory task*: In this task, face stimuli were presented. Half of the faces were presented with direct gaze and half with averted gaze. The task consisted in detecting which face was the same as the face presented two times before. Each target trial consisted of a non-repeated face (with direct or averted gaze) and a repeated face (with direct or averted gaze) that had been repeated two presentations before.	6 Microstates: - MS A: P100 (time window N/A) - MS B: N170 (only present in BPD) (110 – 165 ms) - MS C: N170 (only present in HC) (110 – 165 ms) - MS D: P200 (only present in BPD) (170 – 300 ms) - MS E: P200 (only present in HC) (170 – 300 ms) - MS F: late positive component (present in both groups) (time window N/A) GEV: N/A	- Occurrence	*Occurrence* - MS A: no significant differences between groups - MS B: BPD > HC (p=0.006) - MS C: BPD < HC (p=0.006) - MS D: BPD > HC (p=0.005) - MS E: BPD < HC (p=0.004) - MS F: no significant differences between groups
Berchio et al., 2019 (85)	*2-back gaze working memory task*: In this task, face stimuli were presented. Half of the faces were presented with direct gaze and half with averted gaze. The task consisted in detecting which face was the same as the face presented two times before. Each target trial consisted of a non-repeated face (with direct or averted gaze) and a repeated face (with direct or averted gaze) that had been repeated two presentations before	BD vs HC age-matched: 4 Microstates: - MS 1: P100 (present in both groups) (120 – 160 ms) - MS 2: N170 (present in both groups) (120 – 160 ms) - MS 3: P200 (only present in BD) (180 – 256 ms) - MS 4: P200 (only present in HC) (180 – 256 ms) GEV: N/A Offspring vs HC: 6 Microstates: - MS 1 - 2: P100 (time window N/A) - MS 3: N170 (only present in offspring) (124 – 172 ms) - MS 4: N170 (only present in HC) (124 – 172 ms) - MS 5: P200 (only present in offspring) (160 – 228 ms) - MS 6: P200 (only present in HC) (160 – 228 ms) GEV: N/A	- Occurrence - GEV	**BD vs HC age-matched:** *Occurrence:* - MS 3: BD > HC o For target face with direct gaze (p=0.013) o For matched face with direct gaze (p=0.048) o For marched face with averted gaze (p=0.019) - MS 4: BD < HC o For target face with direct gaze (p=0.004) o For target face with averted gaze (p=0.027) *GEV:* - MS 4: BD < HC o For target face with averted gaze (p=0.019) o For matched face with direct gaze (p=0.043) **Offspring vs HC age-matched:** *Occurrence and GEV* - MS 3/4: no significant *Occurrence* - MS 5: Offspring > HC o For matched face with averted gaze (p=0.027) *GEV* - MS 6: Offspring < HC For matched face with averted gaze (p=0.018)
Chang et al., 2022 (78)	*Auditory P50 clicks paradigm:* The auditory P50 paradigm consists of 80 pairs of clicks of two identical stimuli (S1 and S2). S1 and S2 have a duration of 1 ms. The stimulus interval is 500 ms and the inter-trial interval between pair-stimulus (trials) is variable (between 8 and 12 s). Participants were asked to listen to the stimuli with no response required.	9 Microstates: MS 1 MS 2 MS 3: P50 component MS 4 MS 5: P50 component MS 6: P50 component MS 7: P50 component MS 8: S1-S2 P50 component MS 9: S1-S2 P50 component (time windows N/A) GEV: - FESZ group: 69.32% - UHR group: 71.65% - HC group: 70.62% GEV: N/A	- Order of appearance - Duration - Occurrence - Coverage	*Order of appearance* - S1-P50 sequence of MS UHR and HC: MS 6 → MS 7 → MS 6 → MS 1 FESZ: MS 7 → MS 6 → MS 1 - S2-P50 sequence of MS HC: MS 5 → MS 6 → MS 5 → MS 3 UHR: MS 3 → MS 5. FESZ: MS 5 *Duration* - MS 7: FESZ > HC (p=0.045) *Coverage* - MS 7: FESZ > HC (p=0.018) - MS 8: FESZ > HC (p=0.028) *Occurrence* - MS 7: FESZ & UHR > HC (p=0.012; p=0.022)
Galderisi et al., 2001 (63)	*Visual detection task:* Visual stimuli were presented on a screen either in the center (central condition) or on the right or left side (lateral condition, RVF and LVF respectively). Individuals were instructed to click a button whenever presented with a task-relevant stimulus (i.e. same-name consonant pairs).	Central condition 4 Microstates: - MS1: P1 (59 – 113 ms) - MS2: N1 (117 – 203 ms) - MS3: N2/P2 (207 – 332 ms) - MS4: LPC (336 – 766 ms) Lateral condition 4 Microstates: - MS1:P1 (63 – 125 ms) - MS2: N1 (129 – 250 ms) - MS3: N2/P2 (254 - 390.6 ms) - MS4: LPC (394.5 - 718.7 ms) GEV: N/A	- Topography	**Central condition** MS1, MS2, MS3 PD = HC (no significant) MS4 leftward shift of the positive centroid in PD (p<0.02) rightward shift of the negative centroid in PD (p<0.04) **Lateral condition (for stimuli at right hemifield)** MS1 rightward and posterior shift of the positive centroid in PD (p<0.02, p<0.05) MS2 rightward shift of the positive centroid in PD (p<0.05) leftward shift of the negative centroid in PD (p<0.02) MS3 PD = HC (no significant)
Haartsen et al., 2020 (84)	*Upright-inverted faces task:* Participants watched a series of trials consisting of a fixation stimulus (500 – 700 msec), followed by a face stimulus in upright or inverted (orientation (28 trials/condition, total 168) for 500 msec (randomized order), and a blank screen (350 msec). Participants were instructed to passively watch the stimuli.	*Adolescents group* 5 Microstates: - MS 1 (time window N/A) - MS 2 (100 – 350 ms) - MS 3 (450 – 650 ms) - MS 4 (time window N/A) - MS 5 (time window N/A) GEV: 91% *Adults group* 6 Microstates: - MS 1 (time window N/A) - MS 2 (100 – 350 ms) - MS 3 (500 – 750 ms) - MS 4 (time window N/A) - MS 5 (500 – 750 ms) - MS 6 (time window N/A) GEV: 91%	- Duration - GFP	**Adolescents’ groups:** *GFP* Condition x group interaction effect: - in MS 2, inverted faces >upright faces; this outcome was higher in HC, as compared to ASD (p=0.010) - no significant group or condition interaction effects for the other MS were recorded (p > 0.05) **Adults’ groups:** *GFP* - MS 5 does not appear in ASD, while in HC its GFP was higher in inverted faces, as compared to upright faces (p=0.038) Condition x group interaction effect: - in MS 6, inverted faces >upright faces (more pronounced difference in HC (p =0.026) *Duration* Condition x group interaction effect: - in MS 2, inverted faces >upright faces; this outcome was higher in HC, as compared to ASD (p=0.026) - in MS 6 upright faces > inverted faces; more pronounced condition difference in HC (p =0.026) - no significant group or condition interaction effects for the other MS were recorded (p > 0.05)
Kleinlogel et al., 2007 (75)	*Continuous Performance Test (CPT):* Subjects were instructed to press a button as fast as possible whenever the letter O was followed by an X (Go condition). If the letter O was followed by other letters (A-H, J, K), it required response inhibition (NoGo condition).	5 Microstates: - MS 1 (0 – 120 ms) - MS 2 (140 – 180 ms) - MS 3: P300 (180 – 340 ms) - MS 4 (340 – 460 ms) - MS 5 (120 – 140 ms, 460 – 600 ms) GEV: N/A	- Duration - Onset - Offset - GFP - Topography	**Go-stimuli condition (only MS3 was analyzed)** *GFP* - SCZ<HC (p<0.05) *Onset/Offset/Duration/Characteristics of the topography* - SCZ=HC (p>0.05) **NoGo-stimuli condition (only MS3 was analyzed)** *Onset* - SCZ>HC (p<0.05) *Duration* - SCZ<HC (p<0.01) Characteristics of the topography: NoGo anteriorisation (NGA) - SCZ>HC (p<0.05)
Kochi et al., 1996 (79)	*Visual detection task:* Visual stimuli consisted of two figures displayed simultaneously on a screen at random intervals. Two conditions were employed, each using one type of rare target stimuli (15% probability), two types of rare nontarget stimuli (each 15%) and one type of frequent non-target stimuli (55%). The four types of stimuli were randomly sequenced in one of two conditions. In condition 1, the targets differed from non-targets on only one perceptual dimension: the color (the target was always a blue disk). In condition 2, the targets differed from non-targets on two perceptual dimensions: color and tilt (the target was always a blue disk with a right tilt).	1 Microstate: - MS associated to P300 (300 – 450 ms) GEV: N/A	- Onset - Offset - Duration of MS - Topography - GFP	**Condition 1 (only one perceptual difference in targets)** *Duration* - SCZ<HC (p<0.07) *Onset* - SCZ=HC (p > 0.05) *Offset* - SCZ=HC (p > 0.05) *Latency for maximal GFP* - SCZ>HC (p = 0.005) **Condition 2 (two perceptual differences in targets)** *Duration* - SCZ<HC (p<0.03) *Onset* - SCZ>HC (p < 0.03) *Offset* - SCZ=HC (p>0.05) *Latency for maximal GFP* - SCZ>HC (p<0.05)
Mauriello et al., 2022 (88)	*Delayed face-matching test:* Stimuli were neutral faces with direct or averted gaze. Each unique face had a single eye-gaze direction for the entire task. The task was to identify as quickly and accurately as possible whether a presented face was the same as the one shown two faces before.	6 Microstates: - MS 1: P100 (72 – 124 ms) - MS 2: N170 (124 – 168 ms) - MS 3: P200 (only present in ADHD) (168 – 236 ms) - MS 4: P200 (only present in HC) (direct gaze: 168 – 276 ms; averted gaze: 168 – 232 ms) - MS 5: N250 (236 – 300 ms) (only present in ADHD) - MS 6: N250 (only present in HC) (direct gaze: 276 – 300 ms; averted gaze: 232 – 300 ms) GEV: N/A	- Duration - Correlation of the MS map to the data - Time coverage - GEV	**Direct gaze** **MS4** *Correlation* - ADHD<HC (p=0.009) *Duration* - ADHD<HC (p=0.009) *GEV* - ADHD=HC (p>0.01) *Coverage* - ADHD=HC (p>0.01) **MS5** *Correlation* - ADHD>HC (p=0.008) *Duration* - ADHD>HC (p=0.008) *GEV* - ADHD=HC (p>0.01) *Coverage* - ADHD>HC (p=0.005) **Adverted gaze** **MS4 & MS5** *Correlation* - ADHD=HC (p>0.01) *Duration* - ADHD=HC (p>0.01) *GEV* - ADHD=HC (p>0.01) *Coverage* - ADHD=HC (p>0.01)
Mucci et al., 2007 (81)	*Auditory oddball paradigm:* Two-hundred auditory stimuli were employed and having three different frequencies: 1000-Hz, 3000-Hz and 6000-Hz. The 1000-Hz tone was designated as the target and rare stimuli (n=52), which required the subject to press a button at its occurrence; the 3000-Hz and 6000-Hz tones were designated as frequent standard tones (n=104) and rare-standard tones (n=44). The interstimulus interval varied randomly between 1500 and 2000 ms.	3 Microstates: - MS 1: N100 (time window N/A) - MS 2: P200 (time window N/A) - MS 3: P300 (time window N/A) GEV: N/A	- Amplitude - GFP - Topography	**Standard tones** **MS N100** *Topography* rightward shift of negative centroid in DS (p<0.02) and NDS (p<0.01), as compared to HC **Target tones** **MS N100** *Amplitude* -DS<HC & NDS **MS P300** *Amplitude* NDS<HC & DS *GFP* - NDS<HC (p<0.02) - NDS<DS (p=0.009)
Perizzolo Pointet et al., 2021 (86)	*Face-evaluation task:* Participants evaluated 500 face-avatars that varied along two dimensions of dominance and trustworthiness. Faces of avatars were presented for 600ms on a black background. Then participants were asked to evaluate how the dominant or trustworthy avatar is on a scale from -2 to +2 (for 4 s). Visual stimuli were distributed into four blocks of 125 stimuli, with an alternation of blocks presenting trustworthiness-related avatars only and blocks displaying dominant-related avatars only.	8 Microstates: - MS A: P100 - MS B-F: N170 - MS G-H: Late Positive Potential (time windows N/A) GEV: N/A	- Presence and order of appearance -GEV -Duration	**Dominance-related condition** *Presence of MS* P1: MS-A present in both groups (primary visual perception) N170: MS-B in control group, MS-C in PTSD LPP: only MS-G in PTSD, MS-G -> MS-H in HC *GEV* MS-N170 dominance=1: significant between-group differences (p=0.003) *Duration* MS-H dominance=4: PTSD > control (p=0.032) MS-G dominance=4: PTSD < control (p=0.032) **Trustworthiness-related conditions** *Presence of MS* P1: MS-A present in both groups (primary visual perception) N170: MS-C -> MS-D -> MS-E in PTSD group, only MS B in the control group LPP: only MS-G in PTSD, MS-G -> MS-H in HC *Duration* MS-D trust=4: PTSD < control (p=0.021)
Perrottelli et al., 2023 (64)	*Modified version of the monetary incentive delay (MID) task:* Subjects had to press a button within a predefined time window to win or avoid losing money. A cue stimulus anticipated if the trial was associated with the possibility of gaining money (reward trials) or avoiding losing money (loss trials) or with no effect on the money (neutral trials). After cue presentation (250 ms), subjects waited for a variable interval (delay; 2000 – 2500 ms) and then had to respond to a white target square that appeared by pressing a button. After the target presentation, a feedback appeared (1650 ms), notifying subjects whether they won or did not win money on reward trials or whether they lost or did not lose money on the loss trials.	4 Microstates: - MS 1: three occurrences (0 – 100 ms;100–300 ms; 600 – 700 ms) - MS 2: two occurrences (0 – 150 ms; 150 – 450 ms) - MS 3: two occurrences (150 – 350 ms; 400 – 600 ms) - MS 4: one occurrence (500 – 700 ms) GEV: 83.7%	- Onset - Offset - Duration - AUC - GFP - Center of gravity	**Analysis of the group (SCZ/HC) x stimulus (reward/neutral/loss) interaction effect** **MS1** 1^st^ occurrence: no significant interactions 2^nd^ occurrence: *Duration* - reward HC > neutral HC (p=0.0036) - reward SCZ < neutral HC (p=0.035) - reward HC > reward SCZ (p=0.020) *AUC* - reward SCZ < neutral SCZ (p=0.00020) - reward HC > reward SCZ (p=0.019) - loss SCZ < neutral SCZ (p = 0.039) 3^rd^ occurrence: no significant interactions **MS 2** - 1^st^ occurrence: no significant interactions - 2^nd^ occurrence: *AUC* - reward HC > neutral HC (p=0.024) - reward SCZ > neutral SCZ (p=0.039) - reward HC > reward SCZ (p = 0.031) - loss HC > neutral HC (p=0.022) - loss HC > loss SCZ (p=0.0008) *GFP* - loss HC > neutral HC (p=0.048) - loss HC > loss SCZ (p=0.0050) **MS 3** *1^st^ occurrence*: does not appear in HC group in loss and reward conditions *2^nd^ occurrence:* no significant interactions **MS 4** no significant interactions
Rohde et al., 2020 (42)	*Cue reactivity task.* 28 alcohol-related and 28 neutral trials were interspersed with 28 scrambled trials (in which scrambled pictures served as an additional control condition) and 9 question mark trials that controlled for vigilance. The sequence of trials was pseudo-randomized. The participants indicated the appearance of each stimulus with a button press and had to press another button if a question mark appeared. *Imagination task* Immediately before each cue reactivity task, an individualized imagination task was administered, which was either related or unrelated to alcohol (i.e., neutral). For the alcohol-related imagination task, participants were asked to remember a specific, well-known situation in which they used to drink alcohol. For the neutral imagination task, participants were asked to remember a neutral, everyday situation, e.g., being on the way to work.	7 Microstates: - MS 1: peristimulus component (0 – 68 ms) - MS 2: P1 (70 – 122 ms) - MS 3: N1 (134 – 154 ms) - MS 4: P2 (165 – 190 ms) - MS 5: pre-P3 (218 – 320 ms) - MS 6: P3 (322 – 482 ms) - MS 7: late P3 (484 – 668 ms) GEV: 65%	- GFP - Duration	**MS 2 (P1 component)** *GFP* Group effect: - HC > AUD (p=0.0094) Group x imagined experience x picture interaction: - AUD: no significant effects - HC: alcohol picture > neutral picture (p = 0.007; with an even larger difference between alcohol and neutral pictures after an alcohol-imagined experience, p = 0.004) *Duration* - no significant main or interaction effects **MS 5 (pre-P3 component)** *GFP* - no significant interaction *Duration* Picture effect: - alcohol < neutral (p = 0.0006) Group x picture interaction: - difference alcohol-neutral in AUD > HC (p = 0.0008) Group x imagined experience x picture interaction: - In AUD, difference alcohol-neutral picture after alcohol imagined experience > after neutral imagined experience (p = 0.0114) **MS 6 (P3 component)** *GFP* Picture effect: - alcohol > neutral (p = 0.0002) Group x picture interaction: - difference alcohol-neutral: HC > AUD (p = 0.0046) Group x imagined experience x picture interaction: - HC difference alcohol-neutral picture after alcohol imagined experience > after neutral imagined experience (p = 0.0026) *Duration* *- n*o significant main or interaction effects **MS 7 (late P3 component)** This MS is not present in AUD in response to alcohol-related stimuli after an alcohol-related imagination session
Soni et al., 2019 (80)	*Visuospatial working memory (VSWM) task:* All participants were required to match encoded pairs of identical multi-colored abstract designs hidden in an array. A 4 × 4 array of abstract pictures was presented for 10 s during which the spatial location of the pictures had to be encoded. After 10 s, the pictures were hidden in the array. The matching trial began with a mouse click that exposed a picture for a second and lasted as long as it took to click on another picture chosen as the matching pair. The second picture was considered the response for the trial. In a successful trial, the matched pair of pictures disappeared from the array, while an error trial did not alter the array. The subject had to search for matching pairs of pictures while clicking on them and all pairs of pictures had to be matched correctly to complete the task.	*For Correct trials* - Pre-trial phase: 4 Microstates - Pre-response phase: 6 Microstates (time window N/A) *For Error trials* - Pre-trial phase: 3 Microstates - Pre-response phase: 4 Microstates (time window N/A) GEV: N/A	- Topography - GEV - Time coverage - Duration	**Pre-trial EEG microstates (correct trial) MS1** *Duration* - SCZ<HC (p=0.001) *GEV* - SCZ<HC (p=0.013) *Time coverage* -SCZ<HC (p=0.001) **Pre-response EEG microstates (correct trial)** **MS 4** *Duration* - FDR and SCZ < HC (p=0.041) *GEV* - FDR < SCZ and FDR < HC (p<0.001) *Time coverage* - FDR < HC (p<0.001) **Pre-trial and pre-response EEG microstates (error trial)** - SCZ=FDR=HC for all parameters and MS investigated
Tschuemperlin et al., 2020 (82)	*Alcohol valence task (IAT):* Picture of alcoholic (matched to an individual’s drink of choice) and neutral beverages were paired with positive or negative words. During “alcohol-positive” blocks, participants had to press the same response button for “alcohol” and “positive attributes,” while “water” and “negative attributes” shared the other. In “alcohol-negative” blocks, “alcohol” and “negative attributes” shared 1 response button, while “water” and “positive attributes” shared the other. Only the 160 alcohol trials (alcohol-positive [AP] and alcohol-negative [AN] assignments) were included in the ERP analyses.	11 Microstates: MS 1 (time window N/A) MS 2 (0 – 320 ms) MS 3 (0 – 320 ms) MS 4 (0 – 320 ms) MS 5 (only in AUD) (320 – 700 ms) MS 6 (only in HC) (320 – 700 ms) MS 7 (only in AUD) (320 – 700 ms) MS 8 (320 – 700 ms) MS 9 (700 – 1000 ms) MS 10 (only in HC) (700 – 1000 ms) MS 11 (only in AUD) (700 – 1000 ms) GEV: 93.83%	- Presence and order of appearance - Duration - GFP	*Presence and order of appearance* - MS 1 - 4,8 and 9 (in both groups) - MS 5,7 and 11 (only in AUD group) - MS 6 and 10 (only in HC group) *GFP* - MS8: AUD<HC (p = 0.007) - MS9: congruent > incongruent trials (this difference between conditions was higher in HC, as compared to AUD) (p=0.004)
Zhao et al., 2023 (87)	*Negative emotional stimuli task* The study used emotional face pictures, that included one neutral and eight different negative emotions, selected from the Chinese Facial Affective Picture System. Participants were shown these images randomly (200 trials) and instructed to press a button to discriminate between neutral or negative emotional faces. The MDD-NSSI group repeated the task after 8 weeks of treatment (medication only or medication combined with rTMS).	8 Microstates: - MS 1 - MS 2 - MS 3 - MS 4 - MS 5 - MS 6 - MS 7 - MS 8 (time windows N/A) GEV: 79%	- Duration - Coverage - Occurrence	**Before treatment (MDD, NSSI and HC groups)** **MS 3** *Duration and Coverage* - in negative cues condition: MDD-NSSI > HC (p < 0.05) - in MDD+NSSI: negative cues > neutral cues (p < 0.05) **MS4** *Coverage and occurrence* - in negative cues: MDD-NSSI > MDD (p = 0.002, p < 0.001) **MS6** *Duration, coverage and occurrence* - under negative cues: MDD-NSSI < HC (p = 0.005, p < 0.001, p = 0.007) *Coverage and occurrence* - under negative cues: MDD-NSSI < MDD (p = 0.032, p = 0.022) *occurrence* - MDD-NSSI: negative cues < neutral cues (p = 0.038) **After treatment (only MDD+NSSI groups using negative cues)** **MS 1** *Duration* - med+rTMS < before treatment (p = 0.002) - med+rTMS < med (p = 0.011) **MS 2** *Duration and coverage* - med+rTMS > before treatment (p = 0.001, p = 0.003) - med+rTMS > med (p = 0.011, p = 0.012) **MS 3** *Duration and coverage* - med < before treatment (both p < 0.001) - med+rTMS < before treatment (both p < 0.001) - med+rTMS > med (p < 0.001, p = 0.012) **MS 4** *Coverage and occurrence* - med+rTMS < before treatment (p = 0.001, p < 0.001) - med+rTMS < med (p = 0.002, p < 0.001) **MS 6** *Duration, coverage and occurrence* - med > before treatment (p = 0.001, p < 0.001, p < 0.001) - med+rTMS > before treatment (p = 0.032, p < 0.001, p < 0.001)

ADHD, attention deficit-hyperactivity disorder; AUD, alcohol use disorder; ASD, autism spectrum disorder; AUC, area under the curve; AVH, auditory verbal hallucination; BD, bipolar disorder; BPD, borderline personality disorder; DS, deficit schizophrenia; EEG, electroencephalography; ERP, event-related potential; FDR, first degree relative; FESZ, first episode schizophrenia; GEV, Global Explained Variance; GFP, global field power; HC, healthy control; LPC, late positive complex; LPP, late positive potential; MDD, major depressive disorder; MS, microstate; NDS, non deficit schizophrenia; NH, no hallucination; NSSI, non-suicidal self-injury; PD, panic disorder; PTSD, post-traumatic stress disorder; rTMS, repetitive transcranial stimulation; SCZ, schizophrenia; UHR, Ultra-High-Risk for psychosis.

The number of electrodes used for EEG signal acquisition varied across studies, ranging from 13 to 256 channels (**Table 2**).

Different software tools were used for MS analysis: RAGU (6 studies) (42, 64, 76, 82–84), Cartool (4 studies) (80, 85, 86, 88), Brain Vision Analyzer (2 studies) (75, 77), Microstate EEGlab toolbox (2 studies) (78, 87). Three studies did not specify the software used (63, 79, 81).

Clustering algorithms for MS analysis also varied: Atomize and Agglomerate Hierarchical Clustering (AAHC) (2 studies) (76, 84), K-means (8 studies) (64, 80, 82, 83, 85–88), modified K-means (2 studies) (42, 78), topographic clustering (1 study) (77). Four studies did not specify their clustering algorithm (63, 75, 79, 81).

To determine the optimal number of MS prototypes, studies employed various measures of fit: cross-validation criterion (10 studies) (42, 64, 76–78, 80, 82–84, 87), global explained variance (4 studies) (42, 78, 86, 87), meta-criterion (2 studies) (85, 88), Krzanowski–Lai criterion (1 study) (86), stability and discrimination criterion (1 study) (63). Three studies did not specify the measure of fit used (75, 79, 81).

Parameters extracted from MS varied, with most of the studies considering multiple features (e.g., global field power (GFP), mean duration, GEV, and occurrence) (**Table 3**). More specifically, the MS parameters employed were:

- Amplitude: The magnitude of the EEG signal during a microstate, reflecting the intensity of underlying neural activity.
- Area under the curve (AUC): for a set of time-points assigned to a particular microstate class, the AUC is simply defined as the sum of the global field power (GFP) values of those time-points;
- Center of gravity: is an index that provides information regarding both the magnitude and temporal characteristics of the MS, since it evaluates the distribution of GFP values across the time points assigned to that microstate;
- Correlation of the MS map to the data: the spatial correlation coefficient quantifying the similarity between a microstate template map and the EEG scalp potential distribution at each time point assigned to that specific MS;
- Coverage: the percentage of total recording time that a particular microstate class occupies;
- Duration: the average microstate duration in the sample for a specific MS;
- Global explained variance (GEV): the proportion of the EEG signal’s global variance explained by a microstate class;
- Global field power (GFP): a measure of the spatial standard deviation of the EEG potential field across electrodes at a given time point, reflecting the overall strength of the scalp electric field;
- Occurrence: the average number of times per second a specific microstate appears;
- Onset/Offset: the time point marking the beginning (onset) or end (offset) of a continuous segment assigned to a specific microstate class;
- Order of appearance: the sequential position in which microstate classes emerge during the recording, describing the temporal arrangement or pattern of transitions between microstates;
- Presence of the microstate: it indicates if a specific microstate class was back-fitted to the data of a subject or group;
- Topography: the spatial distribution of electric potentials over the scalp at a given time point or averaged over time, characterizing the stable, class-defining map of a microstate.

Finally, twelve of the included studies explicitly matched some of the topographic maps of the MS to the ERP of interest (42, 63, 75–79, 81, 83, 85, 86, 88) (**Table 3**).

### 3.3 Results of the microstate analysis

**Figure 2:**
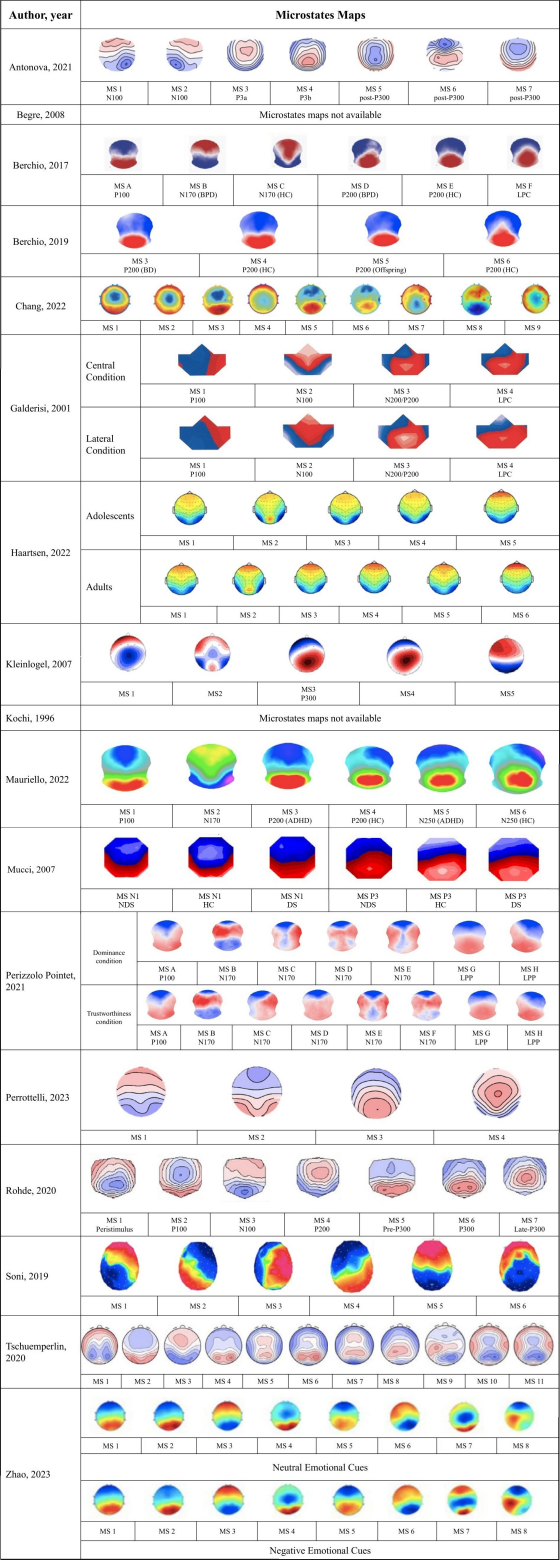
Topography of the microstate maps determined in the included studies. ADHD, attention deficit-hyperactivity disorder; BD, bipolar disorder; BPD, borderline personality disorder; DS, deficit schizophrenia; HC, healthy control; LPC, late positive complex; LPP, late positive potential; MS, microstate; NDS, non deficit schizophrenia. Note: For higher resolution, please refer to the web-based version of this figure.

The following sections report the statistical results comparing study groups (patients vs. healthy controls) of the included studies. The results follow the experimental paradigms used to record ERP data and perform MS analysis. Detailed results are presented in **Table 3**. Due to the variety of tasks employed and the clustering methodology for MS analysis, the characteristics and polarity of the identified microstates were heterogeneous (**Figure 2**). Furthermore, the nomenclatures of the microstates reported in the following sections, tables, and **Figure 2** were taken directly from the original articles. As a result, microstates with the same name or classification (e.g., MS 1 or MS A) do not necessarily share topographic configuration or characteristics across studies. Additionally, two studies (83, 86) labelled microstates alphabetically; however, this alphabetical classification does not align with the canonical microstates commonly identified in resting-state studies.

#### 3.3.1 Auditory processing

Chang et al. (78) studied auditory sensory gating in first-episode schizophrenia (FESZ), individuals at ultra-high risk for psychosis (UHR), and healthy controls (HCs) using an auditory P50 clicks paradigm. Seven microstates were identified without defining specific time windows, and group comparisons focused on their order, duration, occurrence, and coverage. The microstate sequence differed between groups for both auditory clicks (S1 and S2) (**Table 3**). Furthermore, while the first six microstates showed no significant group differences, MS 7 (P50-related appearing after S1) had greater duration and coverage in FESZ compared to HCs, and its occurrence was higher in both FESZ and UHR groups compared to HCs. Furthermore, MS 8, the microstate related to the difference between S1 and S2 clicks, showed a significantly higher coverage in the FESZ compared to the UHR and HCs groups. Mucci et al. (81) studied attention during an auditory oddball paradigm in patients affected by deficit schizophrenia (DS) and non-deficit schizophrenia (NDS). Two microstates were the main focus of the analyses, one related to N100 and one to P300. The authors did not specify the time windows of each MS identified. The analysis focused on the microstate mean amplitude measured at each lead, the GFP, and the topography of maps. Particularly, the N100-related MS recorded both during standard and target stimuli showed a significant reduction in amplitude and a rightward shift of negative centroid in subjects with DS, compared to NDS and HC groups. Conversely, for the P300-related MS, only patients affected by NDS displayed a reduction in GFP compared to DS subjects and healthy controls (81).

#### 3.3.2 Visuospatial processing

Six studies investigated visuospatial processing, examining its sensory and cognitive electrophysiological correlates. Specifically, two studies focused on sensory processing with a visual detection task (63, 79), three examined attention and vigilance (75–77), and one assessed working memory (80).

A visual detection task was employed in two studies. Kochi et al. (79) investigated MS characteristics related to the P300, identified in a time window from 300 to 450 ms from the stimulus, using a visual detection task with two conditions: (1) targets differing by one perceptual feature and (2) targets differing by two perceptual features. In individuals with schizophrenia, MS latency at the point of maximum global field power (GFP) was significantly longer, and MS duration was significantly shorter, as compared to healthy controls (HCs) in both conditions. Galderisi et al. (63) identified four microstates and analyzed their topography under two conditions based on target location. For centrally presented targets, MS 4, associated with the late positive complex (LPC) and identified in a time window between 336 and 766 ms following the onset of stimuli, showed a leftward shift of the positive centroid and a rightward shift of the negative centroid in patients with panic disorder (PD), as compared to HCs. For laterally presented targets, MS 1 (associated with P100; time window: 63–125 ms following the onset of stimuli) showed a rightward and posterior shift of the positive centroid in patients. Additionally, MS 2 (related to N100; time window: 129–250 ms following the onset of stimuli) displayed a rightward shift of the positive centroid and a leftward shift of the negative centroid in patients relative to HCs.

Three studies focused on attention and vigilance using visual stimuli. Antonova et al. (76) identified seven microstates in a 4-choice reaction task (4-CRT) with lateralized stimuli, without identifying specific time windows for each MS. Differences were observed in the duration and area under the curve (76) of microstates of patients with schizophrenia. MS 3 (related to P3a) had a longer duration in patients than HCs, while MS 6 (related to task responses) was significantly shorter. The AUC of MS 1 (related to N100) was smaller in patients, while MS 3 had a higher AUC in patients than HCs. Begré et al. (77) and Kleinlogel et al. (75) both studied MS characteristics during a continuous performance task (CPT) in schizophrenia. Begré et al. found no significant group differences in MS 3 (related to P300) duration, while Kleinlogel et al. reported shorter and delayed MS 3 (in this study as well, associated with the P300) durations following NoGo stimuli in patients, as compared to HCs. Additionally, GFP during Go stimuli was lower in patients, and NoGo anteriorization (NGA) was increased in patients relative to HCs (75). Furthermore, both studies reported the means and standard deviations for each group for peak component-based and microstate-based analyses of temporal features (latency and duration), enabling us to calculate effect sizes using Cohen’s *d* (**Table 4**). However, the calculation of this effect size index showed that the analysis of microstate features had lower effect size values in both studies (75, 77).

**Table 4:** Main results of source analysis and comparisons with peak component analysis.

First Author, year	Effect size comparison between MS and peak component analysis	Analysis method employed and results from source analysis
Antonova et al., 2021 (76)	N/A	N/A
Begré et al., 2008 (77)	**Go Stimuli** *P300 peak latency* Cohen’s d = 0.60 *Duration of P300 MS* Cohen’s d = 0.22 **NoGo Stimuli** *P300 peak latency* Cohen’s d = 0.56 *Duration of P300 MS* Cohen’s d = 0.55	N/A
Berchio et al., 2017 (83)	N/A	**LAURA** **N170 (120 – 165 ms)** BPD > HC cerebral activation: - L superior frontal gyrus (p=0.006) - R inferior frontal gyrus (p=0.004) - L anterior cingulum (p=0.006) - R anterior cingulum (p=0.009) **P200 (170 – 255 ms)** BPD < HC cerebral activation: - R superior frontal gyrus (p=0.010) - R middle temporal gyrus (p=0.009)
Berchio et al., 2019 (85)	N/A	**LAURA** **P200 MS** *Face with direct gaze* - BD<HC cerebral activity in left superior frontal gyrus (p=0.032) and supplementary motor area (p<0.001). - Offspring>HC cerebral activity in the left orbital frontal cortex (p=0.004). *Face with averted gaze* - BD<HC cerebral activity in left somato-sensory cortex (p=0.028), bilateral medial cingulate cortex (L p=0.022, R p=0.024), and left caudate nucleus (p=0.008). Offspring<HC cerebral activity in left insula (p=0.009), right temporal lobe (p=0.019)
Chang et al., 2022 (78)	N/A	***eLORETA*** Cortical endogenous activity of the microstate identified by the P50 component (MS3, MS5, MS6 and MS7) was localized to the right Brodmann 21 area, middle temporal gyrus of the brain and the right Brodmann 11 area, middle frontal gyrus. No difference between patients and controls were analyzed.
Galderisi et al., 2001 (63)	N/A	N/A
Haartsen et al., 2020 (84)	N/A	N/A
Kleinlogel et al., 2007 (75)	**NoGo Stimuli** *P300 Peak Latency* Cohen’s d = 0.94 *P300 MS Duration* Cohen’s d = 0.98	**LORETA** **Go-stimuli condition** - Similar source localization in SCZ and HC - Source strength: SCZ<HC **NoGo-stimuli condition** - Sources in SCZ localized more prefrontally, as compared to HC (central location) - Source strength: SCZ<HC
Kochi et al., 1996 (79)	N/A	N/A
Mauriello et al., 2022 (88)	N/A	***LORETA analysis*** **Direct gaze condition** **P200 (MS3 and MS4)** - ADHD (MS3) <HC (MS4) cerebral activity in left cerebellum (p=0.008) and vermis (p=0.007) **N250** - ADHD (MS5) <HC (MS6) cerebral activity in left posterior cingulum (p=0-009), left calcarine (p=0-005), left lingual (p=0-003), right lingual (p=0-006), left cerebellum (p=0-002), right cerebellum (p=0-007), vermis (p=0-001) **Averted gaze condition** - ADHD=HC cerebral activity in P200 and N250 time windows (p>0.05)
Mucci et al., 2007 (81)	N/A	**LORETA** **N100 (MS 1)** - DS < HC cerebral activity in posterior cingulate and parahippocampal gyrus - NDS = HC **P300 (MS3)** - DS = HC - NDS < HC cerebral activity in the left temporal, right superior frontal, bilateral posterior cingulate, inferior parietal, and supplementary motor areas - NDS < DS cerebral activity in bilateral cingulate, left superior and left middle frontal areas
Perizzolo Pointet et al., 2021 (86)	N/A	**LAURA** **In dominance-related condition during the presentation of “non-dominant” avatars** **N170** - PTSD>HC cerebral activity in limbic regions (right parahippocampal gyrus), right superior frontal gyrus - PTSD<HC cerebral activity in superior temporal gyrus **LPP** in response to “non-dominant” avatars: PTSD>HC activity in the fusiform gyrus **In trustworthiness-related condition during the presentation of “trustworthy” avatars** **N170** -PTSD<HC cerebral activity in the anterior prefrontal cortex, supramarginal gyrus and premotor cortex. **LPP** -PTSD<HC cerebral activity in the left angular gyrus
Perrottelli et al., 2023 (64)	N/A	**sLORETA** **Second appearance of MS1– Reward Condition:** -SCZ < HC activity in the insula, superior temporal gyrus and orbitofrontal cortex of the right hemisphere -SCZ > HC activity in the left prefrontal cortex and medial frontal gyrus **Second appearance of MS2—Loss Condition** -SCZ < HC activity bilaterally in the superior frontal gyrus, motor cortex and middle frontal gyrus -SCZ < HC activity in left parietal and temporal lobules -SCZ < HC activity in right in the cingulate cortex, in the insula and parts of the somatosensory cortex
Rohde et al., 2020 (42)		N/A
Soni et al., 2019 (80)	N/A	**LORETA** **Source differences in Pre-trial (correct trials) time window** **MS 1** - SCZ<HC cerebral activity in the right extra-nuclear, medial frontal gyrus, orbital gyrus, rectal gyrus, superior temporal gyrus and left medial frontal gyrus (p=0-001) - SCZ>FDR cerebral activity in the right anterior cingulate, medial frontal gyrus, orbital gyrus, postcentral gyrus and precentral gyrus (p=0.002) **Source differences in Pre-trial (error trials) and pre-response (correct and error trials) time windows** - SCZ=FDR=HC cerebral activity (p>0.05)
Tschuemperlin et al., 2020 (82)	N/A	**LORETA** **MS8** - AUD=HC cerebral activity (p>0.05) **MS9** - Alcohol negative>alcohol positive cerebral activity in higher superior frontal gyrus activation was driven by patients. HC group displayed higher activation in the bilateral inferior parietal gyrus as well as the left precuneus, insula, and middle frontal gyrus.
Zhao et al., 2023 (87)	N/A	N/A

ADHD, attention deficit-hyperactivity disorder; AUD, alcohol use disorder; BD, bipolar disorder; BPD, borderline personality disorder; DS, deficit schizophrenia; FDR, first degree relative; HC, healthy control; LAURA, Local Autoregressive Average; s/eLORETA, standardized/exact Low-Resolution Electromagnetic Tomography; MS, microstate; NDS, non deficit schizophrenia; PTSD, post-traumatic stress disorder; SCZ, schizophrenia.

Finally, Soni et al. (80) analyzed microstates during a visuo-spatial working memory (VSWM) task in schizophrenia and their healthy first-degree relatives (FDR). Correct trials exhibited four pre-trial and six pre-response microstates, while error trials exhibited three pre-trial and four pre-response microstates. In this study, the authors did not identify specific time windows for each MS. Group differences were primarily observed in correct trials, where MS 1 (pre-trial phase) had significantly lower GEV, time coverage, and time frames in patients compared to HCs. During the pre-response phase, MS 4 showed higher GEV in patients, compared to the FDR group, but a lower number of time frames in patients compared to HCs.

#### 3.3.3 Face processing

Six studies investigated face processing. Berchio et al. (2017), Berchio et al. (2019), and Mauriello et al. studied working memory tasks related to face processing in patients with BPD, BD, and ADHD, respectively (83, 85, 88). Haartsen et al. explored passive face processing in individuals with autism spectrum disorder (ASD) (84). Perrizzolo-Pointet et al. and Zhao et al. examined microstates during facial emotional recognition tasks in women with post-traumatic stress disorder (PTSD) (86) and adolescents with major depressive disorder (MDD) (87), respectively. Despite the use of different face-processing tasks, most studies examined the same ERPs, namely P100, N170, and P200. Furthermore, three studies highlighted differences in the topographic configuration of the P200 microstate in the two study groups (patients and healthy controls).

Berchio et al. (2017) (83) and Berchio et al. (85) used a 2-back gaze working memory task. In this task, participants were presented with face stimuli (direct or averted gaze) and were required to identify the “matched face”, which was identical to the “target face” shown in two trials earlier. In Berchio et al. (2017), six microstates were identified (N.B.: the classification of MS A-F of this study does not correspond to the canonical resting-state microstate classification), including MS B and MS C (N170-related; time window: 110–165 ms following the onset of stimuli) and MS D and MS E (P200- related; time window: 170–300 following the onset of stimuli). Patients with BPD showed a higher occurrence of MS B (N170-related) and MS D (P200-related), but a lower occurrence of MS C (N170-related) and MS E (P200-related), as compared to HCs, regardless of the face stimulus type. In the second study by the same research group (85), four microstates were identified in patients with BD. Significant group differences were found for MS 3 and MS 4 (P200-related; time window: 120–160 ms following the onset of stimuli). Patients demonstrated a higher occurrence of MS 3 and a lower occurrence and GEV of MS 4 compared to HCs. These differences were particularly pronounced for target faces with an averted gaze and matched faces with a direct gaze. In Mauriello et al. (2022) (88), seven microstates were identified during a delayed face-matching test. Group differences were observed for MS 4 (P200-related; time windows: 168–276 ms following the onset of direct gaze stimuli and 168–232 ms following the onset of adverted gaze stimuli) and MS 5 (N250-related). For direct gaze stimuli, ADHD patients exhibited lower correlation and duration for MS 4 but higher correlation and coverage for MS 5, as compared to HCs, with no significant differences in GEV. No significant group differences were observed for each of the microstates for averted gaze stimuli.

One study focused on passive face processing, focusing on adolescents and adults with ASD using an upright-inverted face task (data on children were not considered in the current review (84).

In the adolescents’ group, MS 2 showed higher global field power (GFP) for inverted faces compared to upright faces, but this difference was smaller in ASD than in HCs.

In the adults’ group, MS 2 lasted longer for inverted faces than upright faces, with this difference being greater in HCs than ASD. Furthermore, in the inverted faces condition, MS 5 did not occur in individuals with ASD, whereas HCs showed higher GFP for inverted faces. MS 6 exhibited higher GFP and longer duration for upright faces in HCs than ASD.

Finally, two studies focused on EEG correlates of recognition of emotions. In Perizzolo-Pointet et al. (2022) (86), eight microstates were identified (N.B.: the classification MS A-H of this study does not correspond to the canonical resting-state microstates) in a face-evaluation task where participants rated avatars on dominance and trustworthiness. During the N170 time window, for trustworthy faces, PTSD patients exhibited a sequence of three microstates (MS C → MS D → MS E), while only MS B occurred in HCs. Additionally, higher GEV for MS B was recorded in response to non-dominant faces, while MS D lasted less for trustworthy faces in PTSD patients. During the LPP time window, HCs exhibited a sequence of MS G and MS H for both dominant and trustworthy faces, whereas only MS G was detected in PTSD patients. PTSD patients also showed a longer MS H duration compared to HCs.

In Zhao et al. (2022) (87), eight microstates were identified during a negative emotional stimuli task in adolescents with MDD and non-suicidal self-injury (MDD-NSSI). For negative cues, MDD-NSSI patients had greater coverage and longer duration of MS 3, as compared to HCs. Conversely, MS 6 showed reduced duration, coverage, and occurrence in MDD-NSSI patients. The study also investigated the effects of repeated transcranial magnetic stimulation (rTMS) and medication, which appeared to improve some microstate alterations in MDD patients (see **Table 3** for details).

#### 3.3.4 Reward- and saliency-related processing

Three studies investigated MS characteristics in reward and saliency tasks.

Perrottelli et al. (64) examined reward processing in schizophrenia using a modified monetary incentive delay (MID) task and applied MS analysis to the anticipation stage (related to the ERP elicited after the presentation of cues) of the paradigm. MS 1 (three occurrences; 1^st^ time window: 0–100 ms; 2^nd^ time window: 100–300 ms; 3^rd^ time window: 600–700 ms) showed shorter duration, earlier offset, and smaller AUC and GFP in patients than HCs for reward cues during its second occurrence. MS 3 (two occurrences; 1^st^ time window: 150–350 ms; 2^nd^ time window: 400–600 ms) had lower AUC and GFP in patients compared to HCs in the loss condition during its second occurrence.

Rohde et al. (42) studied alcohol cue reactivity in alcohol use disorder (AUD). MS 2 (P100-related; time window: 70–120 ms following the onset of stimuli) exhibited lower GFP in patients than HCs. MS 5 (pre-P300; time window: 218–320 ms following the onset of stimuli) had a shorter duration for alcohol pictures, as compared to neutral ones, with greater differences in patients. MS 6 (late-P300; time window: 322–482 ms following the onset of stimuli) showed lower GFP for alcohol-related pictures in patients, as compared to HCs.

Tschuemperlin et al. (82) analyzed microstates in implicit association tasks. Six microstates (MS 1–4, 8, and 9) were present in both groups, while MS 5, 7, and 11 appeared only in AUD patients, and MS 6 and 10 only in HCs. The GFP of MS 8 was lower in patients, and MS 9 had greater GFP for congruent trials, with stronger effects in HCs than in AUD patients.

### 3.4 Results of source analysis

EEG source localization aims to identify brain sources underlying electrophysiological data (93). The main findings from studies that utilized source analysis based on microstate analysis are summarized in **Table 4**. Nine studies employed source analysis, six of which used the Low-Resolution Electromagnetic Tomography (LORETA) method (64, 75, 78, 80, 82, 88), while three used the Local Autoregressive Average (LAURA) analysis method (83, 85, 86).

#### 3.4.1 Auditory processing

In Chang et al. (78), the middle temporal gyrus and middle frontal gyrus were identified as the two neuronal regions associated with P50 microstate activity. However, the study did not explore between-group differences in these regions (schizophrenia vs. HCs).

In Mucci et al. (81), the posterior cingulate and parahippocampal gyrus showed decreased cerebral activity in patients affected by DS during the N100-related MS time window. Subjects with NDS showed decreased cerebral activity in left temporal, right superior frontal, bilateral posterior cingulate, inferior parietal, and supplementary motor areas compared to healthy controls and decreased cerebral activity in bilateral cingulate, left superior, and left middle frontal areas compared to DS subjects during the P300-related MS time window.

#### 3.4.2 Visuospatial processing

In Kleinlogel et al. (75), during the P300-related microstate (MS), Go-stimuli activated the same neuronal regions in patients and HCs. However, NoGo-stimuli elicited activation of more anterior regions (prefrontal areas) in patients compared to HCs.

In Soni et al. (80), a reduction in activity within the right extra-nuclear region, medial frontal gyrus, orbital gyrus, rectal gyrus, and superior temporal gyrus was observed in individuals with schizophrenia, as compared to HCs during the MS 1 time window preceding correct trials.

#### 3.4.3 Face processing

In Berchio et al. (2017) (83), increased activation of the left superior frontal gyrus, right inferior frontal gyrus, and bilateral anterior cingulate cortex was observed during the N170 time window (MS B and C), while reduced activation of the right superior frontal gyrus and middle temporal gyrus occurred during the P200 time window (MS D and E) in individuals with BPD compared to HCs.

In Berchio et al. (2019) (85), significant differences were identified during the P200-related microstate time windows (MS 3 and 4). Individuals with BD showed hypoactivation in the left superior frontal gyrus and supplementary motor area for direct gaze faces. For averted gaze faces, reduced activity was observed in the left somatosensory cortex, bilateral medial cingulate cortex, and left caudate nucleus compared to HCs.

In Mauriello et al. (88), hypoactivation in the left cerebellum and vermis during MS 3 and 4 (P200-related) and in the left posterior cingulum, bilateral lingula, bilateral cerebellum, and vermis during MS 5 and 6 (N250-related) was detected in response to direct gaze stimuli in subjects with ADHD, as compared to HCs.

In Perizzolo-Pointet et al. (86), patients with PTSD exhibited hyperactivation in limbic regions and the right superior frontal gyrus, along with hypoactivation in the superior temporal gyrus during MS B-F (N170-related) for non-dominant avatars. Hyperactivity in the fusiform gyrus was observed during MS G-H (LPP-related). For trustworthy avatars, PTSD patients showed hypoactivation in the anterior prefrontal cortex, supramarginal gyrus, and premotor cortex during MS B-F (N170-related) and in the left angular gyrus during MS G-H (LPP-related), as compared to HCs.

#### 3.4.4 Reward- and Saliency-Related Processing

In Perrottelli et al. (64), hypoactivation of the cingulate cortex and orbitofrontal cortex, the insula, and parietal cortex was observed during the MS 1 (reward condition) and MS 2 (loss condition) time windows in patients with schizophrenia, as compared to HCs.

In Tschuemperlin et al. (82), during the MS 9 time window (alcohol-negative stimuli), patients with AUD exhibited significant hyperactivation in the superior frontal gyrus and hypoactivation in the bilateral inferior parietal gyrus, left precuneus, insula, and middle frontal gyrus compared to HCs.

### 3.5 Correlation between microstates and clinical and cognitive characteristics

Six studies examined correlations between features of microstates and clinical or cognitive variables:

Galderisi et al. (63) recorded a significant correlation between Corsi’s Block Tapping Task (CBTT) performance and the left-right coordinate of the LPC microstate positive centroid in the central condition. Poorer performance, indicative of right temporal-hippocampal dysfunction, was associated with a leftward shift of the positive centroid. For the lateral condition, more pronounced topographic abnormalities correlated with a higher frequency of panic attacks.

In Perrottelli et al. (64), during anticipation of rewards, topographic ERP scores for MS 1 and MS 2 correlated with the anticipation of pleasure but not with the severity of negative symptoms.

In Berchio et al. (83), no significant correlation was found between childhood trauma scores and the time frames of MS B, C, D, and E in individuals with BPD.

In Berchio et al. (BD group) (85), significant positive correlations were observed between state-trait anxiety scores, emotional abuse history, and higher GEV values of P200-related microstates.

Haartsen et al. (84) found no correlation between an EEG index representing deviations in microstate features and clinical symptoms such as social responsiveness or ASD symptom severity.

In Mauriello et al. (88), anxiety severity correlated positively with the GEV of MS 6 for direct gaze stimuli. Additionally, the mean duration of MS 4 was negatively correlated with inattention/impulsivity scores.

## 4 Discussion

Microstate analysis identifies and quantifies a limited set of predominant classes of electrophysiological scalp fields that represent key global functional brain states during EEG recordings. Over the past decades, research has increasingly highlighted the potential of microstate analysis in uncovering neurophysiological alterations associated with mental health conditions. While most studies utilizing this approach have focused on resting-state data, there has been a steady rise in studies examining task-related microstates.

This review aims to demonstrate the efficacy of microstates as a tool for analyzing task-related neuronal responses, providing alternative EEG markers for sensory and cognitive processing stages, and highlighting several spatiotemporal alterations in neuronal dynamics across various psychiatric disorders. The studies included in this synthesis address a diverse range of psychiatric disorders, with schizophrenia being the most extensively studied condition. In addition, two studies investigated alcohol use disorder using alcohol-related stimuli to assess craving-related effects (42, 82), while the remaining studies explored other psychiatric disorders.

Compared to peak component analysis, microstates offer several advantages, including the use of qualitative and quantitative parameters related to microstates, the independence from reference-related variability, and broader temporal exploration, capturing rapid and transient neuronal activity (48, 94). For instance, in a study on language production, microstate analysis revealed differences in temporal features of electrophysiological responses based on when the words were acquired (95). These differences were not detected in any parameter of the peak component analysis in the same dataset (95). However, a critical evaluation of the methodologies and results from the studies discussed in the following sections highlights several limitations and challenges that should be addressed to refine future studies. These include methodological variability, the omission of certain key outcomes from microstate analysis in the results sections of some studies, and the need for standardized protocols to enhance reproducibility and comparability across studies.

### 4.1 Methodological heterogeneity in ERP microstate analysis: challenges and future directions

The review revealed substantial heterogeneity across studies regarding EEG preprocessing, microstate mapping procedures, and analysis criteria.

Regarding EEG Preprocessing, high-pass filter settings ranged from 0.1 to 1 Hz, with 1 Hz being the most commonly used. Low-pass filter settings varied from 20 to 100 Hz, with 30 Hz being the most frequently used. Most studies (n = 9) (42, 64, 75–78, 80, 82, 87) used Independent Component Analysis (ICA) to remove artifacts such as eye blinks and movements, ensuring these were not misclassified as microstates.

The methodology for describing temporal changes in spatial brain activity through microstates has evolved significantly. Early studies (1990s–2000s) relied on global map descriptors and Global Map Dissimilarity (GMD) scores to characterize momentary topographies (45, 96). Advances in free-access software have enabled more sophisticated, data-driven approaches (97–99). Most studies (n = 12) (42, 64, 76, 78, 80, 82–88) employed AAHC or k-means clustering algorithms to compute microstates. Previous evidence suggests that both methods yield comparable results (100). Earlier studies often relied on arbitrary criteria (63, 75, 77, 79), while more recent research has used cross-validation (n = 10) (42, 64, 76–78, 80, 82–84, 87) or combined meta-criteria (n = 2) (85, 88) to determine the optimal number of microstates. Studies recommend using reliable data-driven methods to enhance robustness and minimize subjectivity (46).

Temporal smoothing influences microstate analysis results. Of the sixteen studies, only seven (42, 64, 76, 78, 82, 84, 88) systematically reported these parameters, emphasizing the need for consistent reporting practices. However, maps at the moment of phase inversion exhibit low amplitude and high noise, leading to frequent segment changes and, thus, to shorter global durations of the microstates if no smoothing parameters are introduced (46).

Task paradigms varied widely among studies, with only a few employing similar designs. Even within studies using comparable tasks, stimulus type or differences in task design can affect the elicitation of ERPs (101, 102).

A study by Khanna and colleagues showed that microstate features extracted from the same participants were highly consistent across clustering methods and acquisition using a variable number of electrodes (103). Nevertheless, the heterogeneity in several aspects of EEG data preprocessing and methodological approaches to microstate analysis underscores the need for guidelines to enhance cross-study comparability and reduce unnecessary variability. Therefore, establishing clear protocols for EEG preprocessing, microstate mapping, temporal smoothing, and parameter selection would improve the reliability, reproducibility, and generalizability of findings in this field. By addressing these challenges, future research can better leverage the potential of microstate analysis to advance our understanding of task-related brain dynamics in health and disease.

### 4.2 ERP-microstates findings related to task-based paradigms in psychiatric disorders

Despite the variability in the reported features and paradigms employed, the studies included in this review can be grouped into subcategories depending on the main sensory processes or cognitive domains investigated. All studies except one (77) reported significant differences in microstate features between individuals with psychiatric disorders and healthy controls.

#### 4.2.1 Auditory processing

A single study (78) examined auditory processing during a passive listening task, demonstrating electrophysiological alterations both in baseline responses (S1 tones) and in adaptation to repeated auditory stimuli (difference between S1 and S2 tones). Specifically, the microstate MS7, associated with the P50 response after the first auditory click, showed significant differences in duration, occurrence, and coverage in FESZ and UHR groups, as compared to HCs. MS8 coverage, when neuronal responses to S1 and S2 were contrasted, also significantly differed between patients and controls. Furthermore, the sequence of microstates in response to both S1 and S2 stimuli changed remarkably between the three study groups, suggesting that sensory gating defect in the early stages of schizophrenia might be due to a combination of dysfunctions in sensory processing of stimuli at baseline and reduced ability to adapt to redundant auditory stimuli (104, 105). Source localization analysis indicated that the activated brain regions were primarily concentrated in the right temporal lobe. Moreover, the authors argued that microstate features such as duration, occurrence, and coverage enhanced the precision of statistical models for discriminating at-risk individuals and early-stage schizophrenia patients from healthy controls, as compared to peak component analysis of ERPs.

Conversely, Mucci et al. (81) examined electrophysiological alterations during an active auditory oddball task. Microstate analysis showed that alterations in the characteristics of early-N100-related microstates could only be traced in the subjects with deficit schizophrenia, a specific subtype of this disorder characterized by enduring and idiopathic negative symptoms. Conversely, dysfunctions in the P300-related microstates could only be detected in subjects with nondeficit schizophrenia. The results support the hypothesis that patients with nondeficit schizophrenia who have preserved early sensory and attentional processes have abnormalities of the late stages of information processing, suggesting that different clinical presentations are mirrored by distinct electrophysiological alterations (81). Interestingly, although it is a study related to auditory processing, similar topographic maps and alterations in features of microstates related to N100 or P300 in schizophrenia could be traced in other studies that used paradigms related to attention in the visuospatial modality (63, 75, 76).

#### 4.2.2 Visuospatial processing

Six studies (63, 75–77, 79, 80) examined microstates related to visuospatial processing.

Two studies (63, 79) utilized target detection tasks to explore alterations in the spatial configuration of brain activity in panic disorder and schizophrenia.

In the study by Galderisi et al. on target detection (63), group differences were observed between subjects with panic disorder and healthy controls in both early (sensory information extraction) and later (cognitive and task-related processing) stages of visual processing. These findings suggest reduced activation of neuronal networks localized in the left hemisphere during early sensory processing and right hemisphere deficits during later cognitive stages. The authors proposed that these right-hemisphere impairments might hinder the integration of stimulus features with subjective states such as expectancy or certainty. Furthermore, these shifts were correlated with cognitive task performance and the frequency of panic attacks in participants.

Kochi et al. (79) found that individuals with schizophrenia showed altered neuronal activation patterns, as suggested by alterations in the topographic configuration and duration of the P300-related microstate, suggesting a dysfunction in the coordination of visual processing-related brain regions. However, the absence of topographic maps in the study by Kochi et al. limits direct comparison with the findings of the article by Galderisi et al. Furthermore, neither of these studies reports graphically the progression of microstates recorded in the groups of patients and healthy controls, hindering the possibility of detecting the presence of unique temporal microstate patterns in the groups of patients.

Three additional studies investigated visuospatial attention and vigilance.

Antonova et al. (76) reported a reduction in the strength of early microstates MS 1 and MS 2 in individuals with schizophrenia, likely reflecting decreased N100 amplitude in occipital regions. Furthermore, these two maps of N100-related microstates (MS 1 and MS 2) had a topographic configuration like the one (MS 2) recorded in the study by Galderisi et al. and related to the same event-related potential but with clear lateralization of the map, mirroring the characteristics of the target stimuli. During the later stages of stimuli processing, subjects with schizophrenia exhibited a substitution of the later portion of MS 4 (P3b-related) with MS 3 (P3a-related), in contrast to the microstates pattern observed in healthy controls. This finding is consistent with previous reports of reduced P3b amplitude during visual and auditory oddball paradigms in schizophrenia (106, 107). The alterations may reflect a dysfunction in attention networks, resulting in a parietal-to-frontal shift in P3b scalp distribution (108).

Furthermore, the microstate analysis of the study by Antonova et al. revealed additional insights, showing that within the group of patients, MS 3 characteristics varied between individuals with and without auditory-verbal hallucinations, further highlighting the potential of microstate analysis for clinical characterization.

Two studies examined P300-related microstates during the continuous performance task in individuals with schizophrenia, yielding contrasting results since one study (77) found no alterations in microstate features in patients for both Go and NoGo conditions, while the other (75) observed delayed, shorter, and with a more prefrontal configuration in P300-related microstates of patients during NoGo stimuli. The microstate map of the P300-related microstate recorded during the Go condition also seems to correspond to the one present in other tasks employing visual stimuli (42, 76).

The discrepancies may stem from the smaller sample size in Begrè et al.’s study, suggesting that Kleinlogel et al.’s findings might better reflect group differences. However, one prominent limitation of this study is that the authors only reported the sequence of microstates for the Go condition and for one patient, hindering the possibility of comparison in the sequence of microstates between conditions and between the patient and control groups.

Soni et al. (80) found that individuals with schizophrenia showed altered pre- and post-response microstates during a working memory task, particularly reduced occurrences and durations of MS 1. At the same time, changes in MS 4 were observed only in first-degree relatives, suggesting microstates may reflect both clinical and subclinical dysfunctions. However, in this study, the authors also did not report a visual representation of the sequence of the microstates succeeding in the groups of patients with schizophrenia and healthy controls.

These six studies highlight the utility of microstate analysis in uncovering neural alterations related to visuospatial processing, particularly in schizophrenia. By examining shifts in topographic configurations and durations of microstates, these studies provide valuable insights into the neural mechanisms underlying impairments in visual attention, vigilance, and working memory in psychiatric disorders. However, as highlighted by the study by Antonova et al., a visual representation of the microstates sequence divided by the study groups and condition, which was lacking in most of the studies, could facilitate the exploration of the differences in the activation of distinct neuronal networks. Finally, a related aspect that warrants further investigation is that resting-state EEG and event-related microstates, despite exhibiting distinct characteristics, may still show overlaps in certain topographies. For example, resting-state MS C might spatially correspond to maps associated with P300 and, more specifically, to P3b (42, 76, 109). These observations suggest that some resting-state MS topographies may appear in task-based data, particularly in relation to specific ERP components. However, further analyses, such as spatial correlations, would be needed to confirm these assumptions.

#### 4.2.3 Reward- and saliency-related processing

Three studies focused on neuronal responses to salient cues related to either monetary rewards or alcohol-related stimuli.

Perrottelli et al. (64), in patients with schizophrenia, found alterations in subjects with schizophrenia in the microstate related to N100 during reward anticipation, suggesting reduced attention allocation to stimuli associated with pleasant outcomes. These results highlight the presence of electrophysiological alterations that can be detected already during the early stage of processing of “valenced” stimuli, which were not considered in two studies on the same data using peak component analysis, restricting the analysis to EEG waveforms occurring from 200 ms post-stimulus onset (110, 111). Additionally, results from source localization analysis were in accordance with previous fMRI studies suggesting that impairments in reward processing might be due to alterations within areas of motivational circuits and regions linked to cognitive control, such as the orbitofrontal cortex, the cingulate cortex, and the prefrontal cortex (112, 113).

Two studies examined alcohol-related stimuli in individuals with AUD, investigating altered brain responses underlying cue-elicited craving.

The study by Rhonde et al. (42) showed no differences in the sequence of the microstates elicited between the patients and healthy control groups. However, they found that in AUD subjects, discrimination between alcohol and neutral cues began later (P200 time window) compared to controls, who exhibited this distinction earlier (P100 time window). These results highlight aberrant deficits in early sensory gating in patients suffering from AUD, which have not been considered in other studies employing peak component analysis in alcohol-related stimuli, but focusing only on later stages of processing (114, 115).

Although in the study by Tschuemperlin et al. no explicit association between microstates and ERPs was reported for the majority of microstates, MS 2 displayed a topographic map similar to the P100-related microstate, while MS 5 and 6 represented P300-related microstates (**Figure 2**) (82). This study reported distinct topographic patterns in later stages (300 ms onwards) of cue processing in AUD subjects and three distinct microstate maps observable only in patients (82). Finally, differences in late positive potential (LPP)-associated microstate suggested dysregulated emotional processing in AUD patients. Results from sLORETA suggested that differences in this late-occurring microstate might be due to modifications in the activation of the superior frontal gyrus, which has been previously linked to the same task in a study involving healthy participants (116).

Overall, these studies highlight the utility of microstate analysis in characterizing temporal and spatial patterns of neuronal activity associated with reward and saliency processing. Interestingly, the studies also recorded electrophysiological alterations in early time windows that are often not considered in peak component analysis in this type of task. Furthermore, the employment of source analysis allows for drawing parallels with neuroimaging studies, reinforcing the reliability and utility of ERP microstates analysis.

#### 4.2.4 Face processing

Microstate analysis has proven effective in investigating neural processes underlying face perception and processing, as demonstrated by a recent review (71). This approach identifies variations in brain network engagement and strength in individuals with psychiatric disorders. Six studies examined face processing using various tasks.

Haartsen et al. (84) observed differences in early microstates (MS 1 and MS 2, related to P100 and N170, respectively) during inverted face presentations in individuals with autism compared to controls. Reduced inversion effects in autism suggest deficits in the visuospatial processing of faces. Later-stage alterations, particularly in adults rather than adolescents, suggest the recruitment of compensatory mechanisms like increased prefrontal cortex activity to meet cognitive demands. These differences were unrelated to symptom severity, suggesting distinct neural processes underlying autism onset versus symptom expression.

Studies on facial repetition effects and working memory in individuals presenting emotional dysregulation (as in ADHD, BD, BPD) also reported altered microstate topographies. In these three studies, the authors highlighted differences in the topographic maps, displaying how the patients presented slightly different configurations of microstates related to the same ERP.

In BPD, changes in N170- and P200-related microstates suggested global alterations in face detection and structural encoding, linked to dysfunctions in fronto-limbic and medial temporal regions, which have a key role in emotion processing and higher cognitive functioning (83, 117, 118). Similarly, BD patients showed atypical P200 topographies and reduced activation in mirror system regions, impairing social intention perception (85). Finally, Mauriello et al. revealed intact early face-processing (P100, N170) but deficits in later stages (P200, N250) of adults with ADHD, affecting attention allocation and face recognition (88). Furthermore, source imaging results from this study revealed hypo-activations in different areas of the cerebellum, which have recently been linked to social functioning in adults with ADHD (119, 120), and in the posterior cingulate.

Finally, two studies employed paradigms involving the processing of faces with specific emotions. In PTSD patients, altered N170-related microstates during dominance- and trustworthiness-related stimuli indicated changes in threat-related face processing. Dysregulated late-stage microstates (LPP) further suggested generalized emotional processing disruption (86). A study on depression found alterations in different parameters of N250-related microstates during negative-emotion face processing, which normalized following rTMS treatment, suggesting a potential for monitoring therapeutic effects (87).

Overall, microstate analysis reveals common and condition-specific neural patterns in psychiatric disorders during face processing. Furthermore, most of these studies reported microstate maps and sequences between groups, allowing a clear comparison of results obtained in the patients versus healthy control groups. Interestingly, subjects with autism seem to show disruptions of the early stages of face processing, as compared to subjects with ADHD, who displayed alterations mainly at later processing stages. Overall, source analyses consistently showed hypoactivation in frontal, temporal, and limbic regions, alongside hyperactivation in areas like the fusiform gyrus and cerebellum, providing insight into impaired social and emotional processing in psychiatric disorders.

### 4.3 Transdiagnostic and disorder-specific applications and advantages of ERP microstates analysis in psychiatric research

The studies included in this review examined a broad range of clinical populations. In studies involving individuals with schizophrenia, event-related potential microstate analysis provides additional insight into the neurophysiological alterations underlying the deficits consistently reported in auditory and visuospatial processing (76, 81, 121–124). Alterations in the magnitude, timing, and topographic features of ERP-related microstates recorded in these studies, particularly within the 100–400 ms window, may reflect inefficient allocation of neural resources and impaired transitions between functional brain states.

One of the main advantages of ERP microstates analysis, in the reviewed studies, lies in its ability to track dynamic changes in scalp topography with high temporal resolution. This is particularly valuable in paradigms using lateralized stimuli. For instance, in the study by Antonova et al. (2021), this approach enabled the identification of hemispheric asymmetries in early visual components, with reduced contralateral activation over the right hemisphere in schizophrenia (76). These findings demonstrate how ERP microstates can uncover subtle, lateralized brain activity differences that may be missed by conventional analysis of ERPs, offering deeper insight into the spatiotemporal dynamics of sensory and cognitive disturbances in schizophrenia.

Early-stage sensory processing deficits were also evident in two studies using auditory paradigms (78, 81). These confirmed the relevance of investigating early ERPs, such as P50 and N100, which are often considered as reliable electrophysiological markers of abnormal auditory processing in schizophrenia. Deficits in these early responses, observed during both passive and attentional conditions, have been hypothesized to contribute to more complex aspects of symptomatology, including negative symptoms and hallucinations.

Similar patterns in microstate parameters emerged in a study employing a reward anticipation task (64). Alterations were detected during both early and late stages of processing, particularly during stages of reward anticipation and loss-avoidance. The microstate approach proved valuable in this context, revealing differences not only in the amplitude but also in the timing and duration of microstates. This highlights the presence of disruptions in the dynamics of large-scale brain networks in schizophrenia. Therefore, a further advantage of ERP microstates analysis in task-based paradigms is its sensitivity to early time windows, which are often overlooked in traditional ERP studies due to *a priori* selection of analysis intervals.

Finally, two studies demonstrated the potential of microstate analysis to aid in the identification of clinical subtypes within schizophrenia (76, 81). Alterations in microstates linked to early auditory processing were found in patients with deficit schizophrenia, a subtype characterized by primary and enduring negative symptoms, as opposed to non-deficit schizophrenia patients. Similarly, patients with auditory verbal hallucinations showed distinct microstate topographies compared to those without these symptoms, supporting the utility of microstate features in differentiating subgroups within the broader diagnostic category of schizophrenia (76).

The reviewed studies also offer compelling evidence for the utility of ERP microstates analysis in identifying alterations at various stages of face processing, encompassing attentional, affective, emotional, and learning-related components, in mood and neurodevelopmental disorders (71). These findings support the potential of alterations of microstate parameters to reflect shared or disorder-specific neurophysiological mechanisms across conditions.

For instance, distinct patterns of ERP-microstates during face processing were observed in individuals with bipolar disorder, borderline personality disorder, and ADHD, as compared to healthy controls (83, 85, 88). While the specific alterations were not entirely consistent across diagnostic groups, their emergence within overlapping time windows suggests the existence of transdiagnostic neurophysiological alterations linked to impaired face processing.

In individuals with bipolar disorder and those at high risk for the condition, alterations were noted at the P200 stage, which is associated with the processing of facial features. Similarly, subjects with BPD exhibited differences in both the N170 component, linked to face detection, and the P200 component, related to structural encoding. These findings point to atypical neural dynamics underlying the early stages of social information processing. Likewise, in individuals with autism spectrum conditions, passive observation of inverted faces elicited weaker modulation of microstates related to low-level visual analysis and face detection (71, 84). These alterations may reflect early-stage impairments in configural face processing, which are commonly reported in autism. However, the substantial heterogeneity in experimental paradigms and the limited number of studies focusing on conditions such as depression, autism, ADHD, and personality disorders currently limit the generalizability of these findings. Further research with standardized protocols and larger clinical samples is needed to determine whether specific microstate alterations can serve as reliable biomarkers for these disorders.

Finally, the two studies examining alcohol use disorder (AUD) underscore the value of assessing both qualitative and quantitative parameters through ERP microstates analysis (42, 82). The first study demonstrated that individuals with AUD show altered brain dynamics during alcohol cue processing, including impairments in early sensory discrimination and reduced engagement of higher-order cognitive processes (42). These findings point to deficits in both bottom-up and top-down processing streams, with blunted early-stage responses associated with increased subjective craving, and reduced later-stage activation potentially reflecting impaired central nervous system inhibition. Therefore, the microstate-based approach offers a valuable perspective on the temporal unfolding of neural dysfunction in AUD and may contribute to the identification of neurophysiological markers of craving and relapse vulnerability. The second study revealed alterations in both the topographic configuration and strength of ERP microstates during alcohol-related cue processing in patients with AUD (82). These differences were interpreted as reflecting the recruitment of alternative neural resources in response to emotionally salient or cognitively demanding stimuli, consistent with compensatory mechanisms engaged during challenging task conditions.

### 4.4 Limitations of the current literature on ERP-microstates and future directions

The use of ERP microstates analysis offers significant advantages in the study of brain function, enabling the investigation of transient, subtle, and rapid neural dynamics without requiring *a priori* definition of time windows of interest, as shown by some of the included studies. Furthermore, microstate analysis enables the investigation of both quantitative and qualitative electrophysiological alterations in mental disorders (60). Despite the advantages and their potential implications, this review also highlights several limitations within the existing literature.

Notable limitations include the relatively low number of studies employing identical experimental paradigms and the variability in analysis methodologies, which prevent direct comparison of studies and reproducibility of findings. Studies investigating the test-retest reliability of microstate analysis have reported high reliability (125–127); however, most of these studies focused on resting-state data, and only a few investigated event-related microstates using similar experimental paradigms (128, 129).

Additionally, the methods used to extract and analyze the EEG microstates have evolved over time. Among the new methods, k-means clustering has emerged as one of the most widely used approaches and is included as a standard method across various EEG analysis toolboxes (83, 85, 130). While advancements in clustering techniques, such as k-means and hierarchical clustering, have improved microstate identification, there are still controversial issues regarding optimal criteria for microstate identification, such as the number of microstates to identify in similar analysis time windows, which might also depend on the complexity of the task and the number of investigated conditions (109). Further complicating the field, the inconsistent reporting of key parameters—such as temporal smoothing criteria or rejection threshold for short-lived microstates—poses additional challenges.

Future studies should adopt standardized preprocessing and analysis protocols to address these limitations. Establishing open-access repositories for microstate templates, including those related to ERP components, would facilitate cross-study comparisons and promote methodological consistency. As noted in the current review, this standardization would enable a better differentiation of microstate subtypes that might otherwise be conflated within the same ERP component. For example, studies examining P300-related microstates have found that multiple microstates may be identified to characterize the P300 time window. These microstates can be distinguished by their topographic configuration—such as frontocentral versus parietal positivity—and by the specific processing tasks involved (76).

Another critical issue is the lack of essential data and values in the reviewed studies. First, many studies did not provide an estimation of the effect size. Effect size is crucial for assessing the robustness of findings and represents a key input for future research (e.g., power analyses) (131). Furthermore, only two studies reported mean and standard deviations of microstate and peak component analysis, allowing a comparison of the effect size obtained using these two approaches (75, 77). These studies suggested a lower sensitivity of microstate analysis in detecting electrophysiological differences between groups compared to peak component analysis. However, further research is needed to compare the relative strengths and limitations of these methodologies in detecting subtle electrophysiological differences using either peak amplitude or microstate features.

Second, only some of the reviewed studies included figures displaying the topographic configurations of the extracted microstates or the actual sequence of microstates occurring throughout the analyzed time window, separately for patients and healthy controls.

Future studies should focus on calculating the effect size of group comparisons and incorporating visual representations of microstate sequences. These steps will be essential for understanding variations in the spatial and temporal dynamics of microstates across clinical populations, supporting cross-study comparisons, and evaluating the possible advantages or limitations of this approach compared to peak component analysis.

Third, only a few studies have explored the association between EEG microstate alterations and the severity of clinical symptoms, highlighting the potential and the limitations of this research field. Among the six studies (63, 64, 83–85, 88) investigating the above association, findings were heterogeneous, underscoring the complexity of interpreting microstate features in relation to psychopathology. Future research should consider the associations of microstate features with either psychiatric symptoms or cognitive functioning and include larger, transdiagnostic samples to better delineate the role of EEG microstates as potential biomarkers.

Finally, several studies reviewed here have employed linear and distributed inverse solutions to estimate the neural sources underlying microstate activity, thereby enhancing the interpretability of results. However, it is essential to note that identical topographies can arise from different brain generators (46). Therefore, source imaging data should be validated against results from complementary neuroimaging modalities whenever possible.

In summary, while combining microstate analysis with ERPs provides valuable insights into the temporal dynamics of brain activity, the field must prioritize consistency and standardization of the methodological approaches to microstates analysis. In addition, integration with other neuroimaging techniques is essential to advance our understanding of brain dysfunction in clinical populations. This standardization would be possible through the definition of guidelines shared by the scientific community for microstate analysis. As discussed in the current review, these guidelines should provide general recommendations regarding parameters to use for microstate analysis (i.e.: ICA correction for eye blinks, use of temporal smoothing) and data reporting (i.e.: clearly reporting topographic maps of extracted microstates, their onsets and offsets and sequence of occurrence on the group/condition average), but also guidance on the different types of tasks and expected outcomes (number and types of expected microstates to be extracted). The development of such guidelines would help address the current heterogeneity of findings and facilitate more consistent comparisons across future studies.

## 5 Conclusions

This review underscores the potential of microstate analysis as a powerful method for examining global neuronal activity patterns underlying sensory processing and cognitive functions in subjects with psychiatric disorders. Noticeably, some of the studies reported the presence of alterations across different time frames of sensory and cognitive processing, which might be missed in peak component analysis. However, the observed considerable methodological heterogeneity highlights an urgent need for standardized analysis procedures to facilitate cross-study comparisons.

Future studies should also aim to provide detailed and comprehensive data regarding the outcomes of ERP microstate analysis. Addressing these challenges will significantly enhance the utility of microstate analysis in advancing our understanding of electrophysiological correlates of sensory and cognitive processing and their alterations in clinical contexts.

## Data Availability

The original contributions presented in the study are included in the article/supplementary material. Further inquiries can be directed to the corresponding authors.
